# Patient-Level DNA Damage Repair Pathway Profiles and Anti-Tumor Immunity for Gastric Cancer

**DOI:** 10.3389/fimmu.2021.806324

**Published:** 2022-01-10

**Authors:** Shenghan Lou, Yufei Wang, Jian Zhang, Xin Yin, Yao Zhang, Yimin Wang, Yingwei Xue

**Affiliations:** ^1^ Department of Gastroenterological Surgery, Harbin Medical University Cancer Hospital, Harbin, China; ^2^ Department of Thoracic Surgery, Harbin Medical University Cancer Hospital, Harbin, China

**Keywords:** DNA damage repair, gastric cancer, prognosis, tumor microenvironment, chemotherapy, immunotherapy

## Abstract

DNA damage repair (DDR) comprises the detection and correction of alterations in the chemical structure of DNA. The dysfunction of the DDR process has been determined to have important implications for tumor carcinogenesis, malignancy progression, treatment resistance, and prognosis assessment. However, the role of the DDR process in gastric cancer (GC) remains to be fully understood. Thus, a total of 2,019 GC samples from our hospital (Harbin Medical University Cancer Hospital in china) and 12 public data sets were included in our study. In this study, single-sample gene set enrichment analysis (ssGSEA) was used to generate the DDR pathway activity profiles of 8 DDR sub-pathways and identify a DDR pathway signature by combining the DDR sub-pathway gene sets. The DDR pathway profiling’s impacts on the clinical outcomes, biological functions, genetic variants, immune heterogeneity, and treatment responses were analyzed through multidimensional genomics and clinical data. The results demonstrate that the DDR pathway profiling was clearly distinguished between tumor and normal tissues. The DDR pathway profiling reveals patient-level variations, which may contribute to explaining the high heterogeneity of human GC for the biological features and treatment outcomes. Thus, tumors with low DDR signature scores were independently correlated with shorter overall survival time and significantly associated with mesenchymal, invasion, and metastasis phenotypes. The statistical model integrating this DDR pathway signature with other clinical predictors outperforms each predictor alone for predicting overall survival in discrimination, calibration, and net clinical benefit. Moreover, low DDR signature scores were tightly associated with genome stability, characterized by low tumor mutational burden (TMB) and low fractions of genome alteration. Furthermore, this study confirms that patients with low DDR pathway signature scores might not benefit from adjuvant chemotherapy and a monoclonal antibody directed against programmed cell death-1 ligand 1 (anti-PD1) therapy. These findings highlighted that the DDR pathway profiling confers important implications for patients with GC and provides insights into the specific clinical and molecular features underlying the DDR process, which may help to facilitate clinical management.

## Introduction

Gastric cancer (GC) is the sixth most prevalent cancer and the third most common cause of cancer-related death worldwide (1). Due to the lack of early symptoms, the majority of the patients with GC are usually diagnosed at an advanced stage (2). The patients with advanced GC still have a poor prognosis (< 20%) despite several treatment options, including surgical resection, targeted therapy, and chemotherapy (3). Immunotherapy is being explored as adjuvant therapy for patients with advanced GC due to the poor prognosis after standard treatment with conventional chemotherapy.

Immune checkpoint inhibitors (ICIs), especially the programmed cell death-1 ligand 1 (anti-PD-1) antibody dramatically changed the therapeutic management for patients with advanced GC. However, some limitations exist using this treatment, namely its uncertain curative effects, the low objective response rates (ORRs), several adverse effects, and even drug resistance after the initial patient response (4–7). Therefore, a biomarker that can predict the efficacy of ICI therapy is urgently needed to improve the response rate of GC patients to ICI treatment. Currently, some studies are demonstrating that biomarkers, including the combined positive score (CPS), tumor mutation burden (TMB), infection with Epstein-Barr virus (EBV), and microsatellite instability (MSI) (8, 9) may be used to predict the response to anti-PD-1 therapy in GC. Nevertheless, some limitations hinder the clinical potential application of these ICI markers. For instance, the TMB lacks standard and consistent cut-off values (10). Hence, screening other biomarkers for ICI therapy is demanding to better stratify patients and previously identify the patients that would benefit from the immunotherapy.

DNA damage repair (DDR) consists of the detection of alterations and correction in DNA chemical structure (11). Generally, the complete DDR pathway contains 8 core sub-pathways, namely base excision repair (BER), nucleotide excision repair (NER), mismatch repair (MMR), Fanconi anemia (FA) pathway, homology-dependent recombination (HR), non-homologous DNA end joining (NHEJ), direct damage reversal/repair (DR), and translesion DNA synthesis (TLS) (12). The interaction of these DNA repair processes repairs DNA damage accurately and promptly prevents gene distortion, and ensures the cell genome’s integrity (13). Consequently, dysfunction of the DDR process has important implications for tumor formation and malignant progression. In addition, tumors that harbor ineffective DNA repair machinery are likely to exhibit greater genomic instability, which is expected to drive malignant progression and generate more aggressive tumor phenotypes (14). Meanwhile, the cellular efficiencies of these repair processes also play a central role in tumor responsiveness during the treatment of patients with cancer (14). The mechanism of action of many anti-tumor agents is to generate DNA damage or target synthetic lethality. The most marked examples are the hypersensitivities of DDR-deficient tumors to poly (ADP-ribose) polymerase (PARP) inhibitors and platinum-based chemotherapies (15–17). The importance of the DDR process has been highlighted in recent years regarding the development and application of immunotherapy for tumor treatment. The evidence indicates that DDR process disorders may induce a hypermutated phenotype, causing a higher TMB (18, 19). The high TMB could generate more neoantigens, facilitating immune recognition and triggering spontaneous tumor-infiltrating lymphocytes (TIL) infiltration (10, 20). Therefore, taken together these results indicate a strong correlation between the DDR process activity and ICI therapy response.

Human GC is a biologically heterogeneous disease (21), exhibiting a wide range of malignant features and responsiveness to oncologic treatments. This study aims to understand if the biologically heterogeneous of GC disease may be explained at least in part for the different activity levels of the DDR pathway. Thus, a method that successfully quantifies the DDR pathway activity might have broad applications in clinical oncology because it would predict patient prognosis and treatment sensitivity. However, only a few studies have been focused on the activity level of the DDR process, and to what extent it affects GC patients’ clinical outcomes and drug response remain to be unclear.

In this study, the single-sample gene set enrichment analysis (ssGSEA) was performed to indirectly quantify the DDR pathway activity. Moreover, a novel patient-level DDR pathway profiling approach was developed to explore the DDR pathway activity’s impacts on the clinical outcomes, biological features, genetic variants, immune heterogeneity, and drug responses. The results obtained provide a valuable resource to guide both mechanistic and therapeutic analyses of the role of the DDR process in human GC, which has the promise to facilitate clinical management.

## Materials and Methods

### Gastric Cancer Dataset Source

This study includes 268 patients with GC, being studied in terms of demographic information, clinical data, and also tissue samples. The samples were obtained from patients who had undergone gastrectomy as the primary treatment between 2016 and 2019 at Harbin Medical University (HMU) Cancer Hospital in China to construct the HMU-GC cohort (22). Surgical specimens obtained at operation were immediately frozen and stored at -80°C until required. All samples were collected after written informed consent was obtained from the patients. This study was approved by the HMU Cancer Hospital Institutional Review Board. For high-throughput mRNA sequencing, 1 µg RNA per sample was used for library construction with the NEBNext^®^ UltraTM RNA Library Prep Kit for Illumina^®^ and were sequenced with Illumina HiSeq 2000. RNA isolation, library construction, and high-throughput mRNA sequencing were performed by Novogene (Beijing, China). The data were deposited in the Gene Expression Omnibus (GEO) repository (GSE184336 and GSE179252) (22).

Gastric cancer gene-expression data sets were systematically searched in GEO and The Cancer Genome Atlas (TCGA) databases. Data sets with missing follow-up data were excluded. A total of 12 public treatment-naive GC cohorts, namely (TCGA-STAD (Stomach Adenocarcinoma), GSE66229/ACRG (Asian Cancer Research Group), GSE15459, GSE57303, GSE34942, GSE38749, GSE29272, GSE84437, GSE26901, GSE26899, GSE13861, GSE28541) were enrolled for further analysis. The basic information for the 12 pubic datasets is shown in **Table S1**.

### Data Preprocessing

For microarray data, the raw CEL files for the data sets from Affymetrix^®^ were processed using the robust multichip average (RMA) algorithm for background correction and normalization through the affy package (23, 24). The raw data from Illumina^®^ were processed using the limma package (25). For microarray data sets without raw data, the normalized matrix files were downloaded directly. For high-throughput RNA sequencing data from the HMU-GC and TCGA-STAD datasets, raw read count values were transformed into transcripts per kilobase million (TPM) values, which are more similar to those generated from microarrays and are more comparable between samples (26).

The Ensembl gene IDs and probe IDs were converted into an official gene symbol using the biomaRt package (27). Multiple probes (or Ensembl) IDs mapping were collapsed to a single official gene symbol by keeping the probe (or Ensembl) ID with the highest average expression. ComBat algorithm in the sva package was to correct the batch effects from non-biological technical biases among different datasets (28).

### DDR Pathway Curation and Profiles

DDR gene list was assembled from relevant gene lists, including the Molecular Signatures Database (MSigDB) (29), an online catalog of DDR genes from recently published resources (https://www.mdanderson.org/documents/Labs/Wood-Laboratory/human-dna-repair-genes.html). Moreover, the DDR gene list also knowledge-based curation of information on specific DNA repair pathways from other published literature (12, 30–32). Multiple gene sets for the same pathway were combined, being identified a total of 490 DNA repair genes with documented roles in the DDR process. The complete gene list is listed in **Table S2**.

The DDR pathway signature was defined as the sum of the remaining 8 sub-pathways. The GSVA package (33) was used to perform ssGSEA to generate individual patient DDR pathway profiles of normalized enrichment scores (NESs). The ssGSEA NESs were used to construct the DDR pathway profiles, reflecting the activity level of the DDR pathways in each sample.

### Associating DDR Pathway Profiles With Prognostic Features

The samples from the patients with survival time lower than three months were excluded, aiming to enhance the robustness of downstream analyses (34, 35). Univariate and multivariate Cox regression analyses were performed to calculate the hazard ratios (HR) and 95% confidence interval (CI). To compare the survival rates between different groups the Kaplan-Meier survival analysis with log-rank test was used. Moreover, the subsets’ prognostic differences were also compared by the restricted mean survival time (RMST) analysis (36). To study the interaction effect subgroup analyses were performed.

Additionally, the DDR pathway signature was integrated with other independent prognostic factors to generate a composite nomogram for model visualization and clinical application. The nomogram’s predictive value was compared with the TNM staging system by the C statistic. The performance of the composite model was evaluated by the calibration curve, time-dependent receiver operator characteristic (ROC) analysis, and decision curve analysis (DCA) (37).

### Functional and Pathway Enrichment Analysis

Weighted correlation network analysis (WGCNA) was performed using the WGCNA package, which aims to identify the purity-related gene modules (38). The scale-free topology fitting index of 0.85 was set as the threshold to construct the signed weighted gene co-expression network. The minimum co-expression module size was set to 30, and the merge cut minimum module merge cut height was set to 0.25. A biweight midcorrelation coefficient (bicor) > 0.4 and *p-value* < 0.05 were selected as the thresholds to find gene modules significantly associated with the DDR pathway ssGSEA scores.

Gene annotation enrichment analysis was performed using the clusterProfiler package (39). Gene set enrichment analysis (GSEA) was also performed to infer the biological processes related to the DDR process (40). In addition, gene set variation analysis (GSVA) was used (33) to explore the significantly enriched pathways in different subsets. The well-defined “*hallmark gene set*s” were selected for the enrichment analysis (29).

### Genomic Alterations and Mutations

Somatic mutation and copy number variation (CNV) data from the TCGA-STAD cohort were collected using the TCGAbiolinks package (41). The GSE62717 dataset from GSE62254/ACRG cohort was also downloaded for the CNV analysis. Mutation data were analyzed and summarized using the maftools package with the initial removal of 100 frequently mutated genes (42, 43). CNV events were detected by the GISTIC 2.0 approach using the segmented Affymetrix SNP 6.0 microarray data (44).

### Estimation of Infiltrating Cells in the GC Microenvironment

To quantify the infiltration level of stromal and immune cells for each GC sample, the stromal and immune scores were calculated using the ESTIMATE algorithm (45). MCPcounter algorithm was used to quantify the proportions of specific immune and stromal cells in the GC samples in agreement with the guideline for transcriptome-based cell-type quantification methods (46, 47). Moreover, based on the H&E whole-slide images from the TCGA-STAD cohort, a convolutional neural network approach was used to identify the percent values of TILs in digitized H&E-stained tissue specimens (48).

### Estimation of the Potential Chemotherapy Response

The most commonly used chemotherapeutic agents in GC treatment (*i.e.*, 5-fluorouracil, cisplatin, oxaliplatin, capecitabine, paclitaxel, docetaxel, irinotecan, and epirubicin), were selected to predict the chemotherapeutic response. Based on the two public drug sensitivity databases, Genomics of Drug Sensitivity in Cancer (GDSC) (49) and Cancer Therapeutics Response Portal (CTRP) (50), the oncoPredict package (51) was implemented for chemotherapeutic response prediction using ridge regression to estimate the half-maximal inhibitory concentration (IC_50_) for each sample. The prediction accuracy was evaluated by 10-fold cross-validation based on each training set. Default values were selected for all parameters, including the combat algorithm for removing batch effect and the mean value for summarizing duplicate gene expression.

### Estimation of the Potential Immunotherapy Response

RNA-seq data of PRJEB25780 study from 45 GC patients who received the anti-PD-1 therapy were downloaded for analyses (9). The submap algorithm was used to evaluate the expression similarity between the DDR-related subgroups and the patients with different responses (52). Furthermore, the relationship between the DDR-related subgroups and immunotherapy response was predicted using the Tumor Immune Dysfunction and Exclusion (TIDE) web tool (http://tide.dfci.harvard.edu/) (53). Patients with higher TIDE scores have a higher chance of anti-tumor immune escape, thereby exhibiting a lower immunotherapy response rate.

### Statistical Analyses

Mann-Whitney or Kruskal-Wallis tests were used to study the existing differences between groups for continuous variables. Two-sided Pearson’s chi-squared or Fisher exact tests were used to analyze the categorical data. Associations between continuous variables were tested using Spearman correlation analysis. All the statistical analyses performed were conducted using R software, and a two-sided *p-value* was tested. The *p-value* < 0.05 was considered statistically significant. The Benjamini-Hochberg method was applied to control the false discovery rate (FDR) for multiple hypothesis testing.

## Results

### Tumor and Normal Tissues Exhibited Distinct Patterns of DDR Pathway Activity

**Figure 1:**
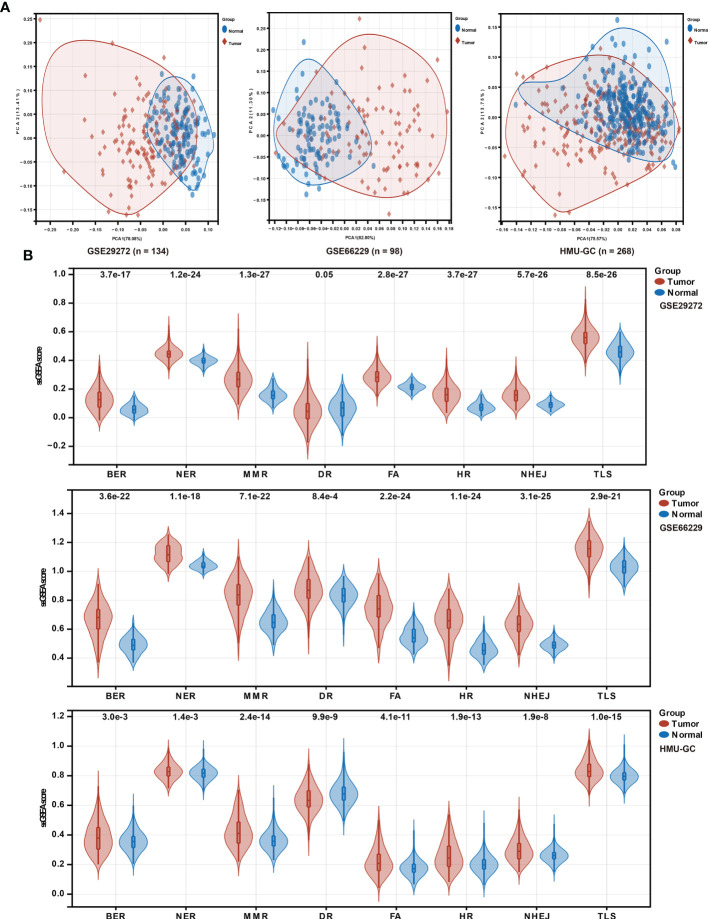
DDR pathway profiling between tumor and normal tissues. **(A)** Principal component analysis (PCA) plots to show distinct patterns of DDR pathway profiling between tumor and normal tissues in the HMU-GC, ACRG, and GSE29272 cohorts. **(B)** Violin plots show the significant differences of DDR pathway profiles between tumor and normal tissues in the HMU-GC, ACRG, and GSE29272 cohorts.

To investigate the putative contribution of the DDR pathway’s activity in GC, in this study ssGSEA method was used to analyze the activation level of the DDR pathway of samples in the HMU-GC, ACRG, and GSE29272 cohorts. Principal component analysis (PCA) determined that the DDR pathway profiles can distinguish with precision between tumor and normal tissues for GC patients (**Figure 1A**). A significant statistical difference was found in the activation level of the DDR pathways between the tumor and normal tissues in each cohort (**Figure 1B**). Apart from the DR pathway, the pathway activity of the other 7 DDR processes was consistently higher in GC tissues. These results confirmed the critical role of the DDR process in GC.

### Correlation of DDR Pathway Profiles With Clinical and Biological Features

**Figure 2:**
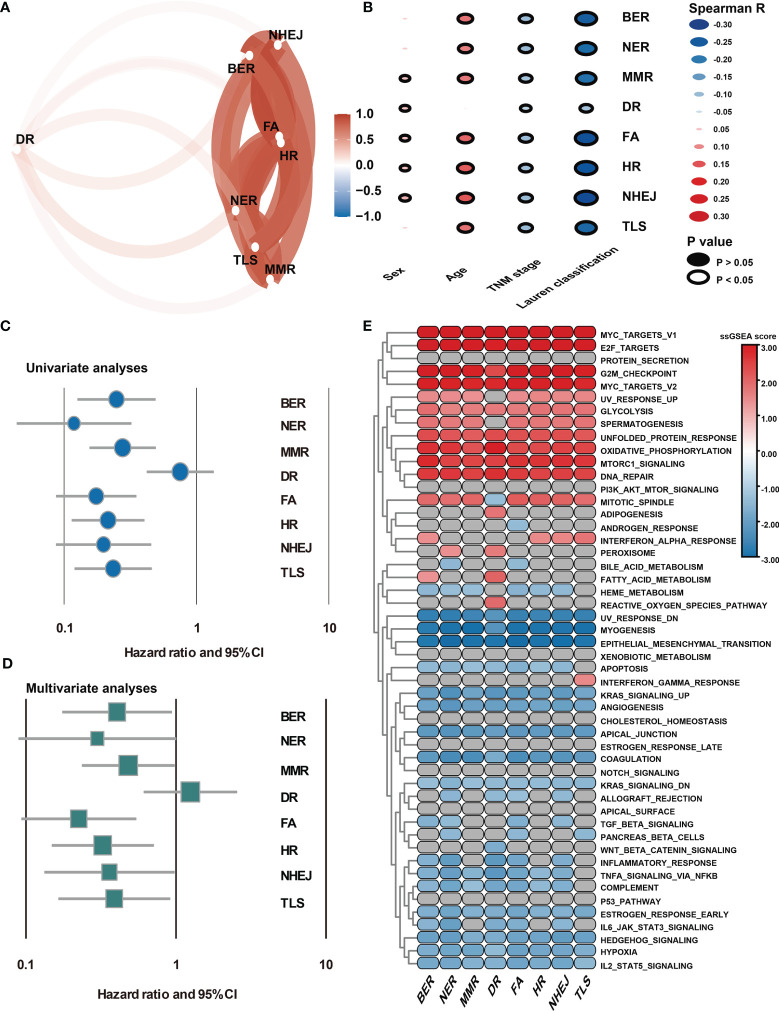
Association of DDR pathway profiles and clinical and biological features. **(A)** The correlation network plot shows the relationship among the 8 DDR sub-pathways. **(B)** Heatmap shows the correlation of DDR pathway profiles and clinical features. **(C)** The Forest plot shows the univariate Cox analysis for the 8 DDR sub-pathways. **(D)** The Forest plot shows the multivariate Cox analysis for the eight DDR sub-pathways, adjusting by age, TNM stage, and the Lauren classification. **(E)** Heatmap shows the GSEA normalized enrichment scores. Grey cells mean non-significant results.

DDR pathway profiling was applied in all the 2,019 patients included in this study. The results show significant correlations among the 8 core DDR pathways (**Figure 2A**). An important result to be mentioned is that the DR pathway gene sets cluster distinctly, indicating that they might capture disparate information. The correlation of DDR pathway profiles was then analyzed with clinical variables (**Figure 2B**). There was a significant but weak correlation with sex for 5 of 8 DDR pathways (Spearman rho [ρ], range, 0.038 to 0.063) and with age for 7 of 8 DDR gene sets (ρ, range, 0.023 to 0.146). The DDR pathway activity was also weakly associated with the tumor, nodes, metastases (TNM) stage (ρ, range, -0.1 to -0.063) and the Lauren classification (ρ, range, -0.275 to -0.087). Thus, DDR pathway profiles were generally weakly correlated with clinical factors, indicating that DDR pathways might provide independent information. Further, the prognostic information that might be contained in the DDR pathway profiles was analyzed. In univariate analysis, 7 of the 8 DDR pathways demonstrated statistically significant associations with a higher overall survival (**Figure 2C**). The age, TNM stage, and Lauren classification were also significantly associated with overall survival for GC patients (**Table 1**). To control for confounders, the individual pathways in multivariate analysis were examined. After adjusting by age, TNM stage, and the Lauren classification, 6 of 8 DDR pathways were found to be significantly associated with these outcomes (**Figure 2D**).

**Table 1:** Univariate and multivariate cox regression analysis of the DDR pathway signature.

	Univariate analysis (n = 1896)		Multivariate analysis (n = 1248)	
	Hazard ratio (95% CI)	*P*-value	Hazard ratio (95% CI)	*P*-value
**Age**				
Increasing years	1.013 (1.007, 1.019)	**<0.001**	1.019 (1.011, 1.026)	**<0.001**
**Gender**				
Female	1 [Reference]			
Male	1.069 (0.929, 1.230)	0.352		
**Lauren classification**				
Intestinal	1 [Reference]		1 [Reference]	
Mixed	1.494 (1.165, 1.914)	**0.002**	1.409 (1.081, 1.837)	**0.006**
Diffuse	1.388 (1.174, 1.642)	**<0.001**	1.289 (1.075, 1.546)	**0.011**
**TNM stage**				
Stage I	1 [Reference]		1 [Reference]	
Stage II	2.016 (1.372, 2.963)	**<0.001**	1.771 (1.181, 2.657)	**0.006**
Stage III	4.268 (3.006, 6.060)	**<0.001**	3.855 (2.664, 5.580)	**<0.001**
Stage IV	8.836 (6.157, 12.679)	**<0.001**	8.001 (5.462, 11.721)	**<0.001**
**DDR pathway signature**				
Increasing values	0.098 (0.040, 0.240)	**<0.001**	0.207 (0.068, 0.630)	**0.006**

CI, confidence interval. The bold values mean that the P value is statistically significant.

Because DDR pathway profiles are closely related to patient prognosis, the biological features associated with the DDR process were analyzed. In this case, the DDR pathway ssGSEA score was correlated with all robustly expressed mRNAs, generating a pre-ranked list sorted by the Spearman correlation coefficient, and performed GSEA (**Figure 2E**). In addition to the DNA repair-related pathways (*i.e.*, DNA_REPAIR, UV_RESPONSE_UP, and UV_RESPONSE_DOWN), some proliferation- and metabolism-related (*i.e.*, E2F_TARGETS, G2M_CHECKPOINT, and GLYCOLYSIS) pathways were significantly activated in GC samples with higher DDR pathway ssGSEA scores. However, invasion- and metastasis-related pathways, such as epithelial-mesenchymal transition (EMT), hypoxia, and myogenesis, were significantly enriched in GC samples with lower DDR pathway ssGSEA scores. Furthermore, the activities of some immune-related pathways (*i.e.*, COMPLEMENT and COAGULATION) were markedly higher in the low-score group than in the high-score group. These findings confirmed that patient-level variation in DDR pathways carried prognostic and biological information, which might be investigated *in vivo* and at the individual patient level for possible use in the clinical management of GC.

### Identification of DDR Pathway Signature

**Figure 3:**
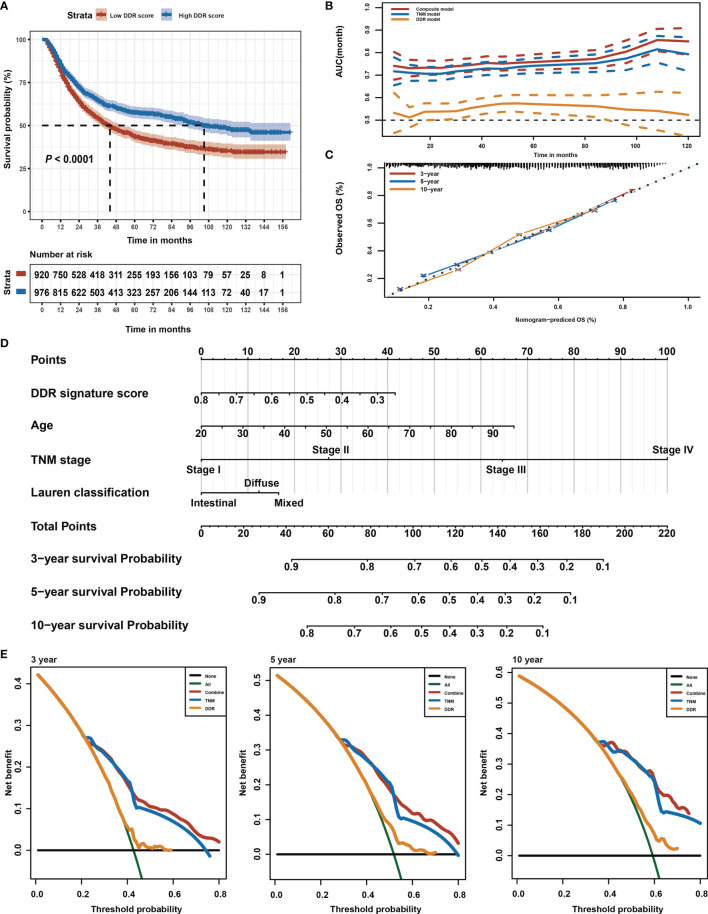
Identification of DDR pathway signature. **(A)** Kaplan-Meier overall survival curve for patients in the low and high DDR signature score groups. The survival difference was detected by log-rank test. **(B)** Time-dependent ROC curves for the composite model, DDR pathway signature model, and TNM classification model. **(C)** Calibration curves of observed and predicted probabilities of the composite model for 3-year, 5-year, and 10-year overall survival. **(D)** Composite nomogram prediction of 3-year, 5-year, and 10-year overall survival. **(E)** DCA curves of the composite model, DDR pathway signature model, and TNM classification model for 3-year, 5-year, and 10-year overall survival.

This study intended to develop a prognostic signature biomarker based on combining the DDR sub-pathway gene sets since DDR pathway profiles are strongly associated with different clinical outcomes and biological functions. This DDR pathway signature was significantly associated with overall survival (**Table 1**). GC patients were further stratified into two subgroups, using the optimal cut-off values (0.525) determined by the X-tile software (54). Kaplan-Meier survival analysis showed that patients with high DDR pathway signature scores had significantly longer overall survival than those with low scores (HR, 0.694; 95% CI, 0.608 to 0.792) (**Figure 3A**). RMST difference also determined a benefit of DDR pathway signature scores at various time points, and the prognostic benefit increases over time (**Table 2**). For example, RMST differences for overall survival between the two subgroups were two months in the third year, five months in the fifth year, and even 14 months in the tenth year.

**Table 2:** Restricted mean survival time (RMST) between different DDR pathway signature subgroups in different time points.

Timepoint	High purity (n = 976)			Low purity (n = 920)			RMST difference^#^			
	RMST	95%CI		RMST	95%CI		Effect size	95%CI		*P*-value
12 months	11.412	11.301	11.524	11.225	11.094	11.355	0.188	0.016	0.359	**0.032**
36 months	29.071	28.379	29.763	26.966	26.209	27.723	2.105	1.079	3.131	**<0.001**
60 months	43.613	42.231	44.995	38.599	37.141	40.058	5.014	3.005	7.023	**<0.001**
84 months	57.169	55.042	59.296	48.512	46.325	50.699	8.657	5.607	11.707	**<0.001**
120 months	75.61	72.314	78.905	61.848	58.51	65.186	13.761	9.071	18.452	**<0.001**

RMST, restricted mean survival time; CI, confidence interval; #, RMST difference = RMST_high DDR signature score_ - RMST_low DDR signature score._ The bold values mean that the P value is statistically significant.

All relevant clinical variables were included in a multivariate analysis and the results point that this DDR pathway signature was independent of standard clinicopathological variables (**Table 1**). Subgroup analyses were performed according to age, sex, Lauren classification, and TNM stage to explore the interaction effect between the DDR pathway signature and clinical factors. No significant interaction effect was observed (**Table 3**), indicating that the DDR pathway signature is a robust prognosticator, retaining its prognostic relevance even after consideration of classic clinicopathologic features.

**Table 3:** Subgroup analysis for the DDR pathway signature among different clinical features.

	Samples	Hazard ratio (95% CI)	*P-*value for interaction
**Gender**			
Female	636	0.048 (0.010, 0.233)	0.299
Male	1260	0.131 (0.044, 0.391)	
**Age**			
< 65	1071	0.078 (0.023, 0.266)	0.943
≥ 65	820	0.075 (0.019, 0.292)	
**Lauren type**			
Intestinal	716	0.192 (0.040, 0.912)	0.763
Mixed	143	0.252 (0.012, 5.125)	
Diffuse	473	0.099 (0.019, 0.515)	
**TNM stage**			
Early (Stage I and II)	537	0.061 (0.006, 0.602)	0.178
Advance (Stage III and IV)	849	0.371 (0.116, 1.181)	

CI, confidence interval.

To support the clinical potential use of these findings, a nomogram was constructed for predicting overall survival based on the DDR pathway signature and other clinical prognostic variables (**Figure 3D**). The composite nomogram (C statistic, 0.695; 95% CI, 0.673 to 0.716) achieved significant improvement for assessing overall survival than the TNM classification (C statistic, 0.675; 95% CI, 0.654 to 0.696), which further increased the C statistic by 0.02 (95% CI, 0.008 to 0.028). The time-dependent receiver operating characteristic (ROC) curve also showed that the composite nomogram had a larger area under curve (AUC) value than the TNM classification and DDR pathway signature for predicting GC prognosis (**Figure 3B**). The calibration curve detected an optimal prediction between the nomogram prediction and actual observations (**Figure 3C**). A good calibration was obtained as well as discrimination for the composite model.

Finally, the net clinical benefit of the composite nomogram was compared with that of the other two models through DCA curves. The composite nomogram demonstrated a larger net benefit in comparison with the TNM classification and DDR pathway signature within most of the above threshold probabilities (**Figure 3E**), indicating that the composite nomogram had better clinical utility for predicting overall survival in patients with GC.

### Biological Insights of the DDR Pathway Signature

**Figure 4:**
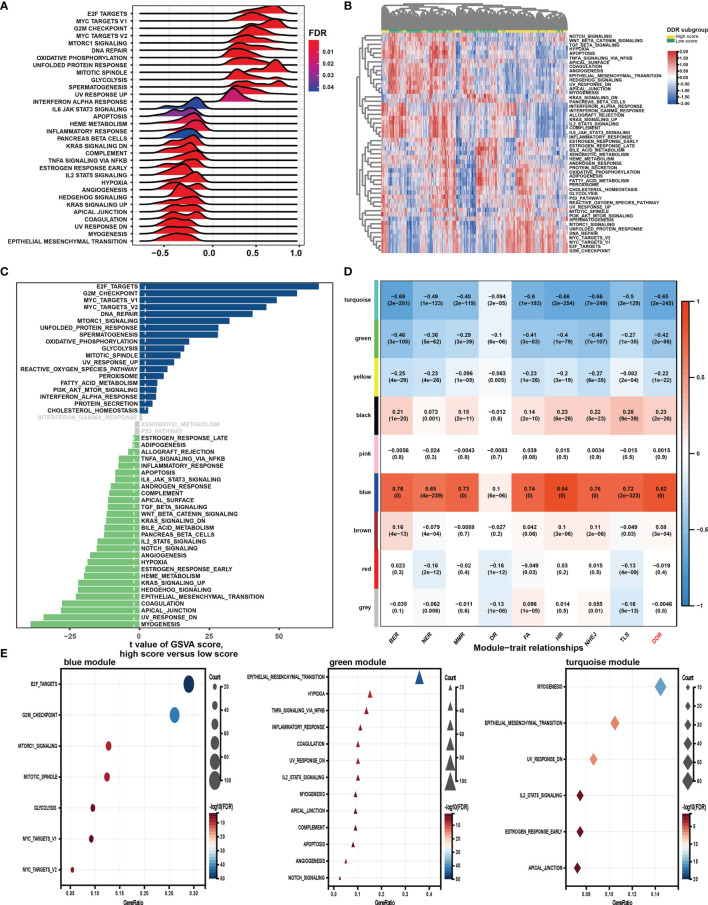
Function analysis of genes correlated with the DDR pathway signature. **(A)** Gene set enrichment analysis (GSEA) of the hallmark gene sets for the DDR pathway signature scores. **(B)** Heatmap shows gene set variation analysis (GSVA) enrichment scores of the hallmark gene sets. **(C)** The bar plot shows the different analysis outcomes for GSVA scores of hallmark gene sets between the high and low DDR score groups. **(D)** Module-trait relationships. Each row shows a module eigengene; each column corresponds to a clinical trait. Each cell contains the corresponding correlation (upper number) and *p-value* (lower number). **(E)** Functional enrichment analysis of the hallmark gene sets for genes in the blue, green, and turquoise modules.

GSEA was first performed to explore the possible mechanism of the DDR pathway signature (**Figure 4A**). The results indicated that pathways related to DNA repair, proliferation, and metabolism hallmarks were significantly enriched in GC samples with higher DDR pathway signature scores. In contrast, stromal and immune-related pathways were activated considerably in GC samples with lower DDR pathway signature scores. Further, GSVA confirmed significant differences in biological functions between the high-score and low-score groups (**Figures 4B, C**). Moreover, some DDR-related, proliferation-related, and metabolism-related pathways were found to be significantly activated in the high-score group compared with the low-score group. However, the activation level of some stromal and immune pathways was markedly higher in the low-score group than in the high-score group.

Additionally, WGCNA was used to obtain the DDR pathway signature-related modules. The top 5000 most variant genes, measured by the median absolute deviation (MAD), were selected for the WGCNA. The cluster dendrogram was constructed according to the optimal soft threshold power of 8 (**Figure S1A**), and 9 color modules were identified (**Figure S1B**). Genes that could not be included in any module were placed in the grey module and removed. Later, the eigengene of the selected traits and modules to evaluate the module-trait relationships were then correlated. Three modules (*i.e.*, turquoise, green, blue) were highly significantly associated with the DDR pathway signature (|R| > 0.4) (**Figure 4D**). Gene significance significantly correlated with module membership in each module (**Figure S1C**), suggesting that genes in these modules might play essential biological roles related to the DDR process.

Module enrichment analyses were further performed to explore the DDR pathway signature-related modules’ functional features (**Figure 4E**). Genes in the blue module were significantly enriched in the proliferation- and metabolism-related pathways, which is in full agreement with the above results. The top enriched terms for genes in the green and turquoise modules were involved invasion, metastasis, and immune response. These findings implied that the DDR pathway profiles reflected the expression alterations of genes involved in GC’s multiple vital hallmarks.

### Genomic Mutations and Alterations Underlying the DDR Pathway Signature

**Figure 5:**
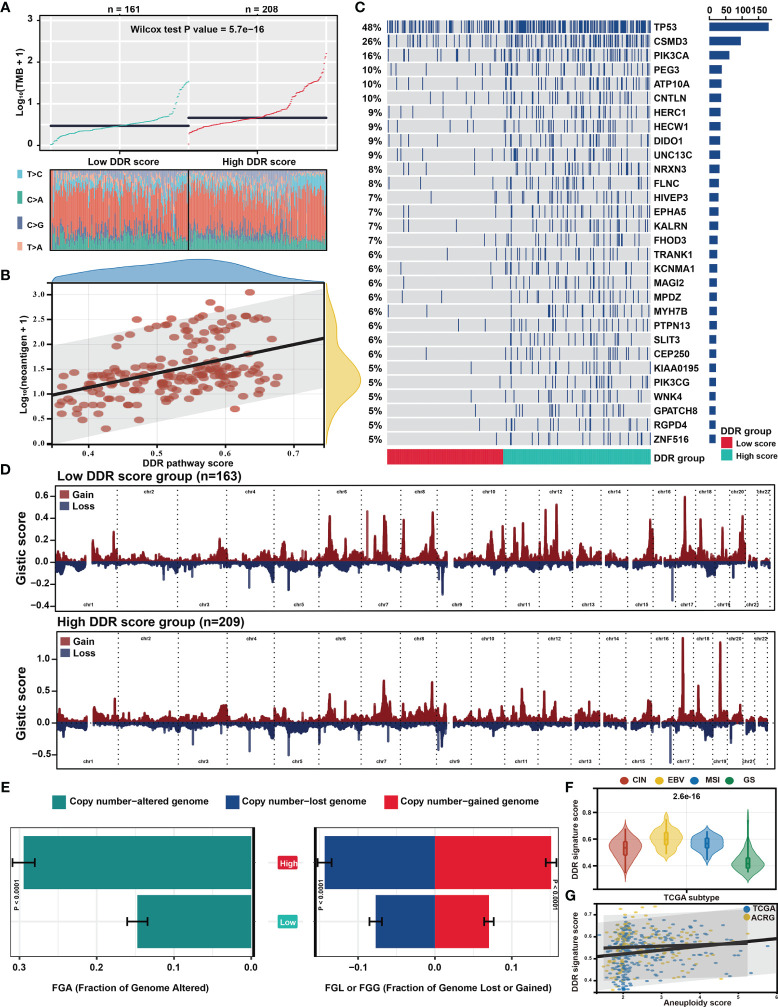
Distinct genomic features associated with the DDR pathway signature. **(A)** The scatter plot shows the different distribution of tumor mutational burden (TMB) in the high and low DDR pathway signature score groups. **(B)** The scatter plot shows the correlation between the DDR pathway signature scores and neoantigen counts. **(C)** The mutational landscape between the high and low DDR score groups in the TCGA cohort. **(D)** The distinct copy number variation (CNV) profile between the high and low DDR score groups in the TCGA cohort. The vertical axis represents the GISTIC score of chromosomal deletion (blue) and amplification (red). **(E)** Bar plots show the different fractions of the genome altered, genome lost, and genome gained between the high and low DDR score groups in the TCGA cohort. **(F)** Violin plots show the different distribution of the DDR pathway signature scores in the four TCGA subtypes. **(G)** Scatter plots show the correlation between the DDR pathway signature scores and the aneuploidy scores in the TCGA and ACRG cohorts.

Genomic data, including mutation profile and somatic copy number alteration (SCNA) data from the TCGA-STAD dataset, were first analyzed to explore the possible genomic features underlying the DDR pathway-related prognostic signature. A significantly higher TMB was detected in the high DDR signature score group (**Figure 5A**). Since more mutations often caused more neoantigens (55), we also determined that neoantigens positively correlated with the DDR pathway signature scores (**Figure 5B**).

Regarding the mutation frequencies, after filtering out genes with low-frequency mutations (*i.e.*, less than 5% of all GC samples), 244 significantly mutated genes were found between the two subgroups (all FDR < 0.05) (**Table S3**). As shown in the oncoprint plot (**Figure 5C**), most genes, including *TP53*, *CSMD3*, *and PIK3CA*, were found to be significantly more mutated in the high-score subgroup. In contrast, the mutation rates of *CDH1* were more frequent in the low-score subgroup.

The CNV data were then investigated, showing distinct chromosomal alteration patterns between the low-risk and high-risk groups in the TCGA cohort (**Figure 5D**). A significantly higher fraction of genome alteration, both the fraction of genome lost and genome gained, was detected in the high-score subgroup than in the low-score subgroup (**Figure 5E**). The distinct chromosomal alteration patterns were further confirmed by the data from the ACRG cohort (**Figure S2**).

The association between the DDR signature and chromosome instability was further investigated since the dysfunction of the DDR process is closely related to chromosome instability (11), The TCGA classification showed that the genomically stable (GS) subtype was characterized with the lowest DDR pathway signature scores (**Figure 5F**). Moreover, the aneuploidy scores also significantly correlated with the DDR signature score in the TCGA and ACRG cohorts (**Figure 5G**). These results suggested that the high activity level of the DDR pathway was strictly related to genome and chromosome instability.

### Association of DDR Pathway Signature and Immune Microenvironment

**Figure 6:**
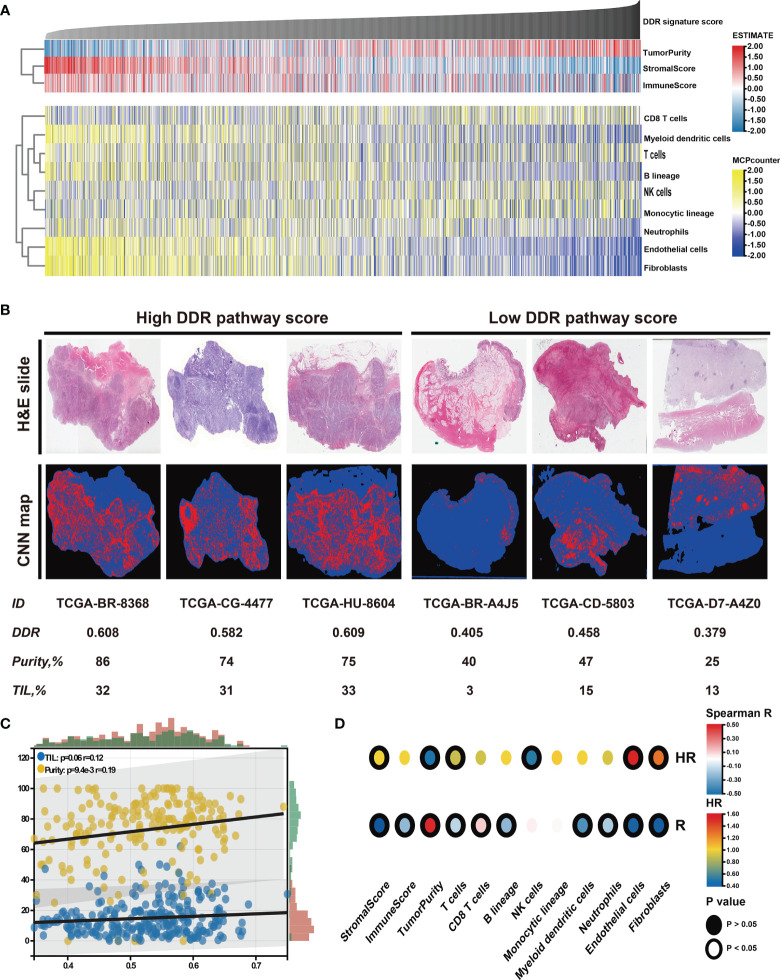
Identification of the tumor microenvironment features underlying the DDR pathway signature. **(A)** Heatmap shows the infiltration level of stromal and immune cells against the DDR pathway signature scores. **(B)** Representative slides (top) and tumor-infiltrating lymphocyte (TIL) maps (bottom) of GC tissues between high and low DDR pathway signature score groups. Red represents a positive TIL patch, blue represents a tissue region with no TIL patch, while black represents no tissue. **(C)** The scatter plot shows the correlation of the DDR pathway signature scores with TILs and tumor purity. **(D)** Heatmap shows survival outcomes of tumor microenvironment features (top) and their correlation with the DDR pathway signature scores (bottom).

The relationship between the tumor microenvironment (TME) status and the DDR pathway signature to characterize their immune heterogeneity was investigated since GC cases with lower DDR pathway signature scores were markedly enriched in stromal and immune activation pathways. The results demonstrate that both the stromal and immune scores, representing respectively, stromal and immune cell infiltration in tumor tissue were inversely correlated with the signature scores and were significantly higher in the low-score group (**Figures 6A**, **S3A**). These results strongly suggested that patients with a higher DDR signature score were characterized by a high level of tumor purity (**Figure S3A**). Contrastingly, patients with decreased signature scores had a significantly increased stromal and immune cells infiltration. Later, the MCPcounter algorithm was applied, aiming to gain insight into the relative abundances of stromal and immune infiltrating cell subpopulations against the DDR pathway signature (**Figure 6A**). Consistent with the ESTIMATE outcomes, fibroblasts, and endothelial cells, representing stroma components, exhibited a consistent negative correlation (*ρ* < -0.4) with the DDR pathway signature score. Meanwhile, most immune cells, such as T cells, B cells, neutrophils, and myeloid dendritic cells, also showed a significant negative association with the signature scores. However, cytotoxic T cells were positively correlated to the DDR pathway signature scores. In addition, significant differences for most TME-related cells were detected between the two subgroups (**Figure S3B**). Based on the pathology whole-slide images, the positive relationship between the tumor purity and the DDR pathway signature score was confirmed (**Figures 6B, C**). Consistent with the MCPcounter outcomes, samples with high signature scores also had a higher percentage of TILs (comprised primarily of cytotoxic T cells and NK cells) than those with low signature scores (48).

Finally, the prognostic implications of these microenvironment features in GC were also investigated (**Figure 6D**). Survival analyses showed that high tumor purity was associated with a good prognosis. For the specific cells, fibroblasts and endothelial cells were consistently associated with worse overall survival. Nevertheless, T cells and NK cells were consistently associated with better overall survival. Taken together, these results indicate that stromal components and the activated oncogenic pathways based on the proposed DDR pathway signature likely contribute to the worse prognosis in patients with low DDR pathway signature scores.

### Potential Response to Chemotherapy and Immunotherapy

**Figure 7:**
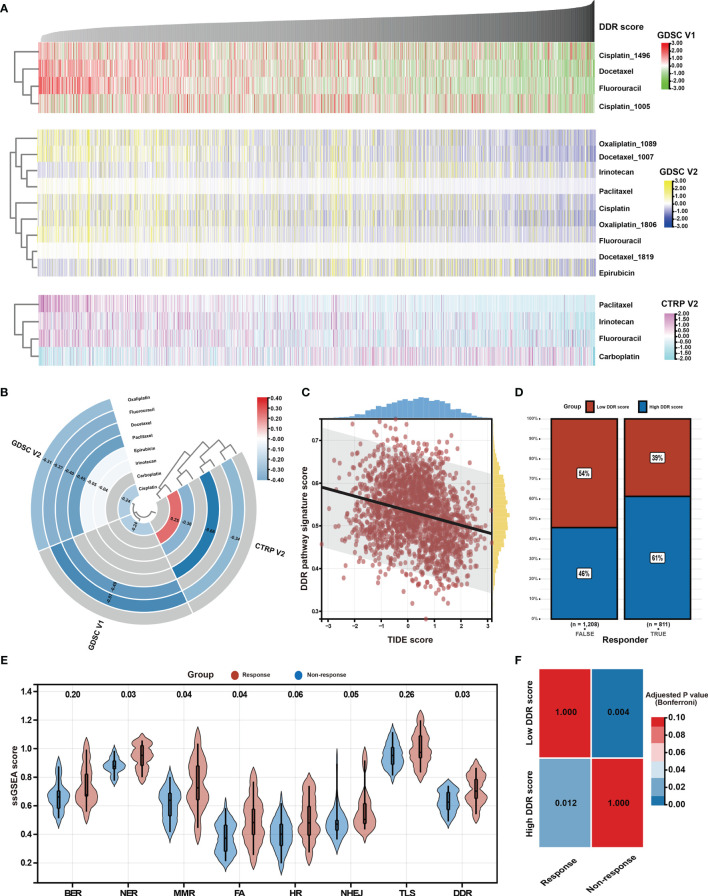
Identification of potential response to chemotherapy and immunotherapy. **(A)** Heatmap shows each patient’s predicted IC_50_ values of the selected chemotherapeutic agents based on GDSC V1, GDSC V2, and CTRP V2 databases. **(B)** Heatmap shows Spearman’s coefficients for the correlation of the DDR pathway signature scores and the predicted IC_50_ values of the selected chemotherapeutic agents. **(C)** The scatter plot shows the correlation between the DDR pathway signature scores and the TIDE scores. **(D)** The bar plot shows the distribution of the DDR pathway signature scores in the response and non-response groups. **(E)** Violin plots show the different distribution of the DDR pathway profiles in the response and non-response groups. **(F)** Heatmap shows the high DDR pathway signature score could be more sensitive to the anti-PD-1 therapy (Bonferroni-corrected *p-value* = 0.012).

Chemotherapy is the main treatment option for patients with advanced GC (56). However, chemotherapy resistance is the primary cause of treatment failure. Given the clinical, biological, and microenvironmental features varied against the DDR pathway signature scores, the relationship between the identified DDR pathway signature and chemotherapy response was investigated, aiming to promote personalized chemotherapy regimens. First, ridge regression was conducted to predict the drug susceptibility outcomes (IC_50_ value) for each sample. The analyses were performed using, respectively GDSC- and CTRP-derived drug response data (**Figure 7A**). Two approaches were used to identify the association of IC_50_ values and DDR pathway ssGSEA scores. First, Spearman correlation analysis was performed to select chemotherapeutic agents with a significant correlation coefficient (|R| > 0.2, *p-value* < 0.05) (**Figure 7B**). Second, differential analysis among the high and low DDR pathway signature score subgroups was conducted to identify drugs with lower estimated IC_50_ values in each subset (**Figure S3**). These analyses yielded 5 GDSC-derived compounds (*i.e.*, fluorouracil, cisplatin, oxaliplatin, paclitaxel, and docetaxel) and 4 CTRP-derived compounds (*i.e.*, fluorouracil, carboplatin, paclitaxel, and irinotecan). Except for carboplatin, all the other drugs had lower estimated IC_50_ values in the high DDR score subgroup and a negative correlation with the DDR signature scores. These results suggest resistance to chemotherapy of these drugs in GC patients with low DDR signature scores.

As above-described, the DDR pathway signature score presented a significantly positive correlation with TMB and neoantigens load (**Figures 5**), which suggests that the patients with high DDR signature scores might benefit from ICI treatment. In full agreement with the idea, a significant negative association was found between the TIDE scores and the DDR pathway signature scores (ρ, -0.260; 95% CI, -0.302 to -0.218) (**Figure 7C**). Besides, the TIDE algorithm determined that patients with high DDR scores (47.38%, 497/1049) might be more likely to respond to immunotherapy than those with low DDR scores (32.37%, 314/970) (odds ratio [OR], 0.532; 95% CI, 0.441 to 0.640) (**Figure 7D**). According to the response to treatment, GC patients in the PRJEB25780 cohort were divided into responder (complete or partial response, CR or PR) and non-responder (stable or progressive disease, SD or PD) groups. The DDR pathway signature scores were significantly higher in the responder group (**Figure 7E**), indicating that patients with a high DDR signature score might show better anti-tumor immune response. The activation of other DDR-related pathways, such as NER, MMR, and FA, might also improve patient response to ICI therapy. The submap algorithm further confirmed that the high DDR pathway signature scores group was more likely to respond to anti-PD-1 treatment (Bonferroni-corrected, *p-value* = 0.012) (**Figure 7F**).

## Discussion

The high-throughput sequencing technology offers an unprecedented view into tumor biology and the promise of personalized healthcare. Unbiased expression researches have been used to identify prognostic and predictive biomarkers in the form of individual signatures (57–59). However, these studies generally waste a large amount of signaling pathway knowledge that has been accumulated over decades of academic research (60). And even more important is that most gene signatures are not being widely used in clinical practice. This may be explained by reproducibility concerns stemming from computer-based algorithms that do not incorporate biological rationale during gene selection (32). In these published studies, the biological importance of the selected genes is retrospectively discussed rather than prospectively examined. Several hypothesis-driven attempts have been made to develop biomarkers or signatures with guidance from pathway knowledge to improve reproducibility (14, 32, 60). Notwithstanding, this approach has not been pursued in GC, in which long disease outcome intervals determine that few cohorts with the most meaningful outcome, overall survival, are available.

The current study presents a hypothesis-driven pathway profiling approach in a large GC meta-cohort with follow-up long enough to acquire robust outcomes. A study flow chart is displayed in **Figure S5**. The approach proposed in this study is based on a well-established bioinformatics algorithm, *i.e.* ssGSEA, providing a novel method to build a pathway-based signature. This study focused on DDR pathways because of their central role in genome stability and potential response to chemotherapy and immunotherapy. The DDR pathway profiles determined were clearly distinguished between tumor and normal tissues. Indeed, there was a significantly higher ssGSEA pathway score in the tumor group than in the normal group. This is consistent with the fact that tumors tissues have higher DDR process activity than normal samples. Moreover, these DDR pathway patterns are less correlated with the clinical factors (*e.g.*, sex, age, TNM stage, and Lauren classification), indicating that the DDR pathway profiles could provide additional information independent of the clinical variables.

Most individual sub-pathways (7 of 8) and the identified DDR pathway signature have a statistically significant prognostic association with overall survival, even after adjusting the clinical variables. These findings suggested that the DDR pathway profiles are an excellent resource to improve the prognosis prediction for patients with GC. This study found that GC patients with a high activation level of the DDR pathways often characterized good clinical outcomes. The results were in agreement with a previous study in urothelial carcinoma (61). Then, a composite model was constructed by combining the DDR pathway signature with other clinical predictors. This statistical model outperforms each predictor alone in discrimination, calibration, and net clinical benefit. A nomogram to simplify its use was then built.

By integrating multidimensional genomics, the DDR pathway profiles also yielded interesting tumor biology insights into malignant progression. Specifically, tumors with low DDR pathway signature scores were enriched in pathways correlated with invasion and metastasis, such as EMT, myogenesis, angiogenesis, and hypoxia. However, proliferation and metabolism pathways (*e.g.*, G2M checkpoint, MYC targets, and oxidative phosphorylation) were significantly activated in tumors with high ssGSEA scores. Notably, TMB and neoantigens showed a positive correlation with the DDR pathway profiles (**Figure 5**). A high degree of genomic instability, especially chromosome instability, was detected in tumors with high DDR pathway signature scores. This finding might partially explain the poor prognosis of patients with low signature scores since the GS subtype was associated with the worst prognosis among the four TCGA molecular subtypes (62). This study elucidates that only *CDH1* mutation rates were more frequent in the low signature scores subgroup. *CDH1* mutation leads to the aberrant expression of *E-cadherin*, disturbing the normal cell and cell adhesion, thus contributing to the metastasis and invasion phenotypes in tumors (63).

Tumors characterized by high genome instability and large numbers of neoantigens often had a higher degree of proinflammatory activity in comparison with tumors without these extensive changes (64–66). Therefore, tumors with different signature scores might have different immune microenvironment statuses (**Figure 6**). A previous study suggested that TP53 mutations were associated with higher levels of leukocyte infiltration (67). Accordingly, increased levels of TLS, especially infiltration of CD8+ T cells, were related to the high DDR signature scores. The increased lymphocyte infiltration usually means a superior prognosis and a potent anti-tumor immune response (68), indicating the immune-hot status in tumors with high signature scores. However, tumors with low DDR pathway signature scores were enriched with fibroblasts and endothelial cells, together with the TGF-β signaling pathway activation, corresponding to an immune-cold phenotype (69, 70).

Looking beyond the clinical and biological implications of the DDR pathway profiles, the results presented herein might also have important inferences to personalized medicine. A previous study suggested that GC patients with the chromosome instability (CIN) subtype most benefitted from adjuvant chemotherapy. However, GC patients of the GS subtype are resistant to chemotherapy (62). Moreover, the tight correlation between the DDR pathway signature scores and TMB and neoantigens load suggested the activation of the DDR pathway could serve as a biomarker for ICI treatment (10), predicting the efficacy of anti-PD-1 therapy in patients with GC. In good agreement with previous observations, high DDR pathway signature scores could identify tumors that respond to PD-1 blockers and hypersensitivity to specific classes of chemotherapeutic agents (**Figure 7**), indicating that the DDR pathway signature could potentially be used to guide the oncologic treatments that are best suited for individual patients.

The present study demonstrates the specific clinical translational value of patient-level DDR pathway profiles in GC. To the best of our knowledge, this is the first study that investigated the role of DDR pathway activity in a large GC cohort. Compared with previous studies that mainly focused on mutations of a given gene in the DDR pathway (12), this study was based on the complete signaling pathway from the view of the transcriptome, which provides a new perspective. Considering that the high costs and technical challenges may limit the use of whole-genome sequencing in clinical practice, clinical management from the overall activation level of the DDR pathway has unique advantages.

Although pathway profiling represents a novel approach, this study also has some limitations that need to be mentioned. Thus, the exact meaning of pathway ssGESA score is ambiguous and is not a direct marker of pathway activity. Moreover, there may be a significant discrepancy between DDR gene expression and enzymatic activity, but a high-throughput analysis of enzymatic activity is not yet possible. On the other hand, there are also limitations of the retrospective cohorts, which do not have complete phenotypic data, randomized treatment, or uniform follow-up. Since this study was mainly based on bioinformatics and statistical analyses, the *in silico* outcomes still need *in vivo* and *in vitro* validations. In the future, corresponding cell experiments and animal experiments will be performed to determine the influence of the activation level of the DDR pathway in GC, aiming to confirm the outcomes presented in the present study.

## Conclusions

This study identified a novel patient-level DDR pathway profiling approach that revealed distinct DDR pathway clusters and demonstrated that the DDR pathway profiles have broad implications for cancer biology and oncologic patient care of GC. With additional prospective validation in clinical and mechanistic studies, the identified DDR pathway signature could become a powerful tool to be useful in stratifying advanced-stage GC patients toward personalization treatments incorporating chemotherapy and immunotherapy.

## Data Availability Statement

The datasets presented in this study can be found in online repositories. The names of the repository/repositories and accession number(s) can be found in the article/**Supplementary Material**.

## Ethics Statement

The studies involving human participants were reviewed and approved by Institutional Review Board of the Harbin Medical University Cancer Hospital. The patients/participants provided their written informed consent to participate in this study.

## Author Contributions

YX and SL designed this work. SL, YFW, JZ, and XY integrated and analyzed the data. SL, YFW, and JZ wrote this manuscript. SL, YZ, and YMW edited and revised the manuscript. All authors approved this manuscript.

## Funding

This work was supported by funding from the Project Nn10 of Harbin Medical University Cancer Hospital (Grant Number Nn102017–03).

## Conflict of Interest

The authors declare that the research was conducted in the absence of any commercial or financial relationships that could be construed as a potential conflict of interest.

## Publisher’s Note

All claims expressed in this article are solely those of the authors and do not necessarily represent those of their affiliated organizations, or those of the publisher, the editors and the reviewers. Any product that may be evaluated in this article, or claim that may be made by its manufacturer, is not guaranteed or endorsed by the publisher.

## Supplementary Material

Supplementary Figure 1 Weighted correlation network analysis (WGCNA) for the DDR pathway signature-related gene modules. **(A)** Identification of the soft threshold according to the standard of the scale-free network. The red line represents the threshold line of 0.85. **(B)** Hierarchical dendrogram of the co-expression modules identified by WGCNA. **(C)** Intra-modular analysis for the signature-related modules. The scatterplot shows gene significance *vs.* module membership in the blue, green, and turquoise modules.

Supplementary Figure 2 Copy number variation (CNV) profile underlying the DDR pathway signature. **(A)** Distinct CNV profile between the high and low DDR score groups in the ACRG cohort. The vertical axis represents the GISTIC score of chromosomal deletion (blue) and amplification (red). **(B)** Bar plots show the different fractions of the genome altered, genome lost, and genome gained between the high and low DDR score groups in the ACRG cohort.

Supplementary Figure 3 Tumor microenvironment features underlying the DDR pathway signature. **(A)** Violin plots show the different distribution of stromal score, immune score, and tumor purity between the high and low DDR signature score groups. **(B)** Violin plots show the different distribution of stromal and immune cells infiltration levels between the high and low DDR signature score groups.

Supplementary Figure 4 Potential response to chemotherapy. Violin plots show the different distribution of the IC_50_ values for the selected chemotherapeutic agents in the high and low DDR signature score groups based on **(A)** GDSC V1, **(B)** GDSC V2, and **(C)** CTRP V2 database.

Supplementary Figure 5 The research workflow of this study.

Supplementary Table 1 The basic information of samples in the 12 public datasets.

Supplementary Table 2 A total of 490 genes annotated as DDR process-related factors and acquired from published resources.

Supplementary Table 3 Gene mutation rates between the high and low DDR signature score groups in the TCGA cohort.
