# Supplementary figures and images for: Patient-Level DNA Damage Repair Pathway Profiles and Anti-Tumor Immunity for Gastric Cancer
Source: Front Immunol. 2022 Jan 10;12:806324. doi: 10.3389/fimmu.2021.806324 (PMC8785952; doi:10.3389/fimmu.2021.806324)

A

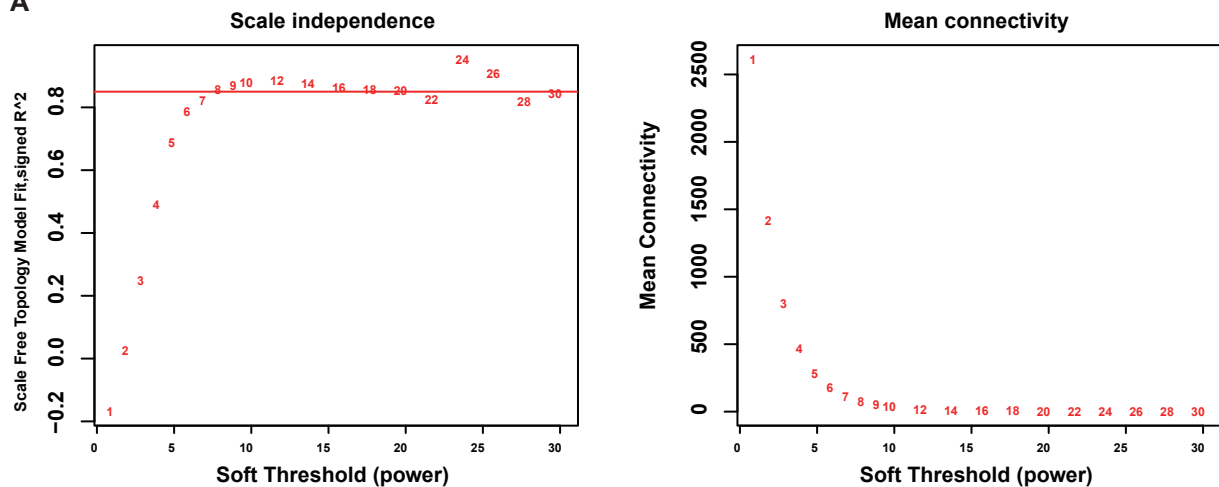

B

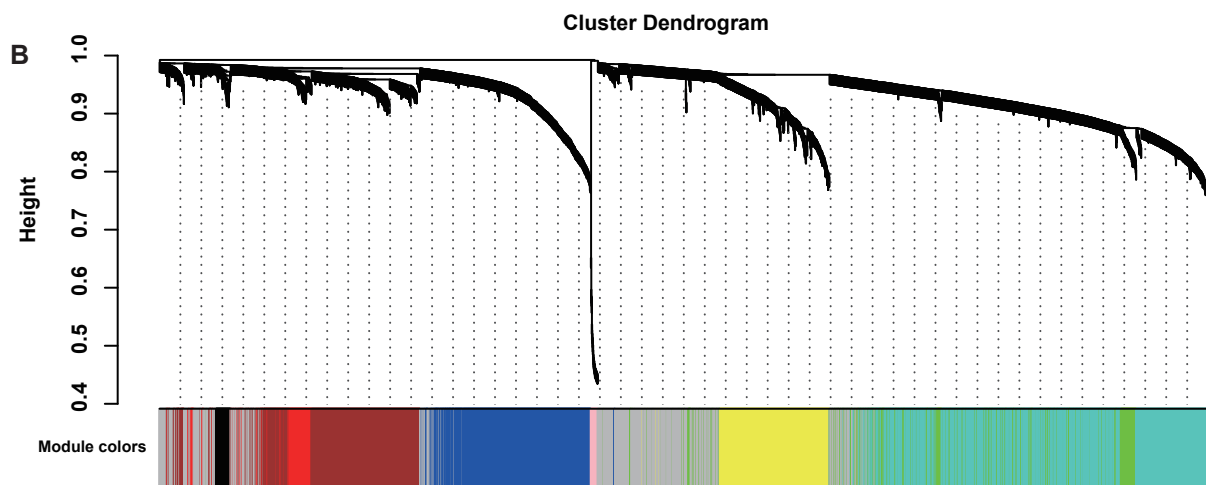

C

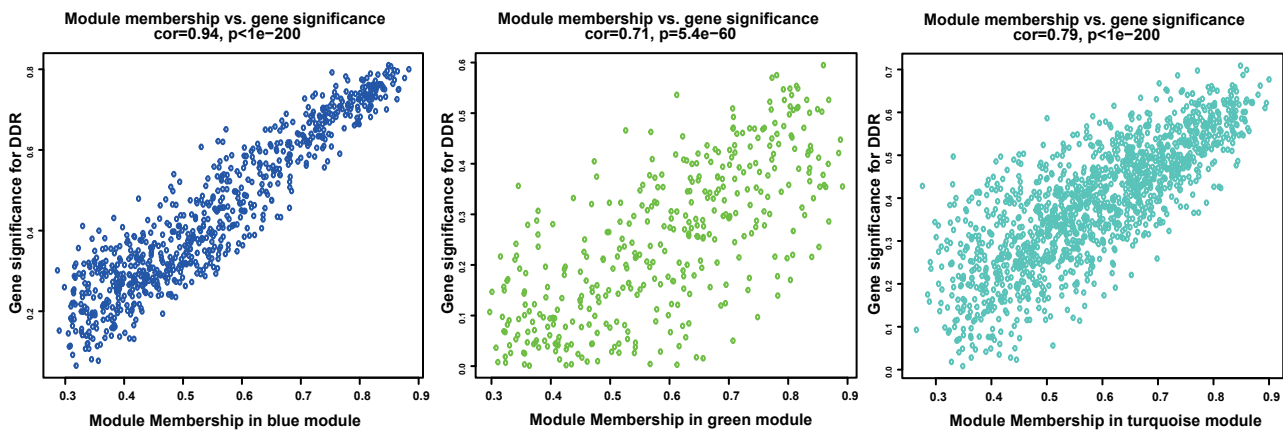

Supplement: Supplementary Figure 1 — Weighted correlation network analysis (WGCNA) for the DDR pathway signature-related gene modules. (A) Identification of the soft threshold according to the standard of the scale-free network. The red line represents the threshold line of 0.85. (B) Hierarchical dendrogram of the co-expression modules identified by WGCNA. (C) Intra-modular analysis for the signature-related modules. The scatterplot shows gene significance vs. module membership in the blue, green, and turquoise modules. [file Image_1.pdf]

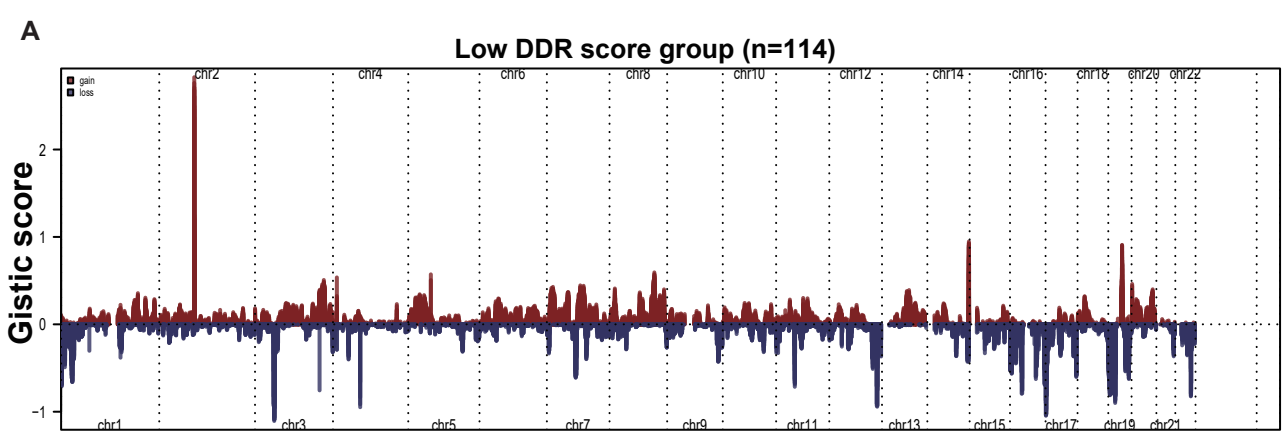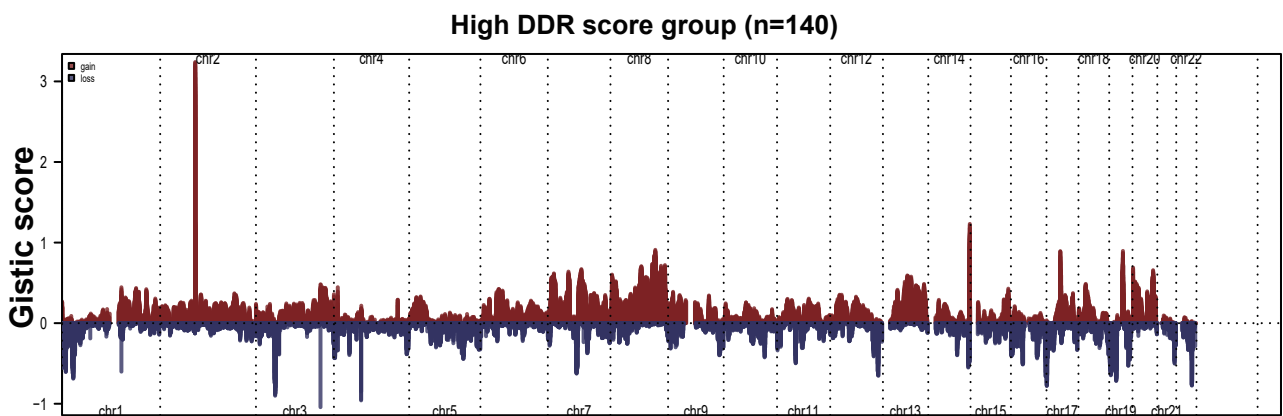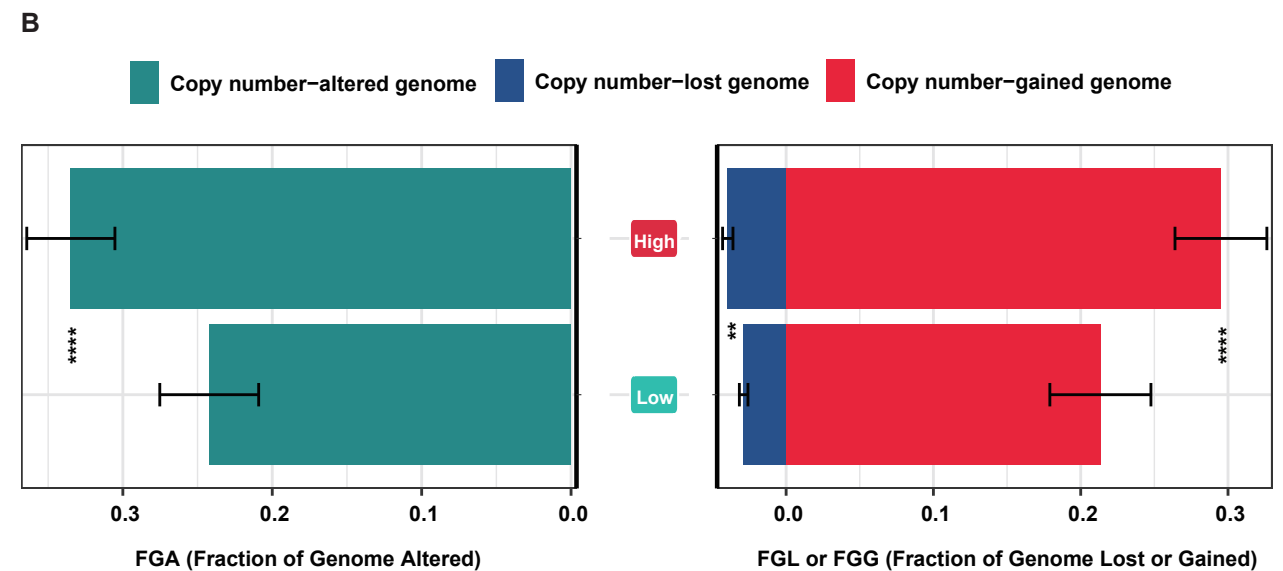

Supplement: Supplementary Figure 2 — Copy number variation (CNV) profile underlying the DDR pathway signature. (A) Distinct CNV profile between the high and low DDR score groups in the ACRG cohort. The vertical axis represents the GISTIC score of chromosomal deletion (blue) and amplification (red). (B) Bar plots show the different fractions of the genome altered, genome lost, and genome gained between the high and low DDR score groups in the ACRG cohort. [file Image_2.pdf]

**A**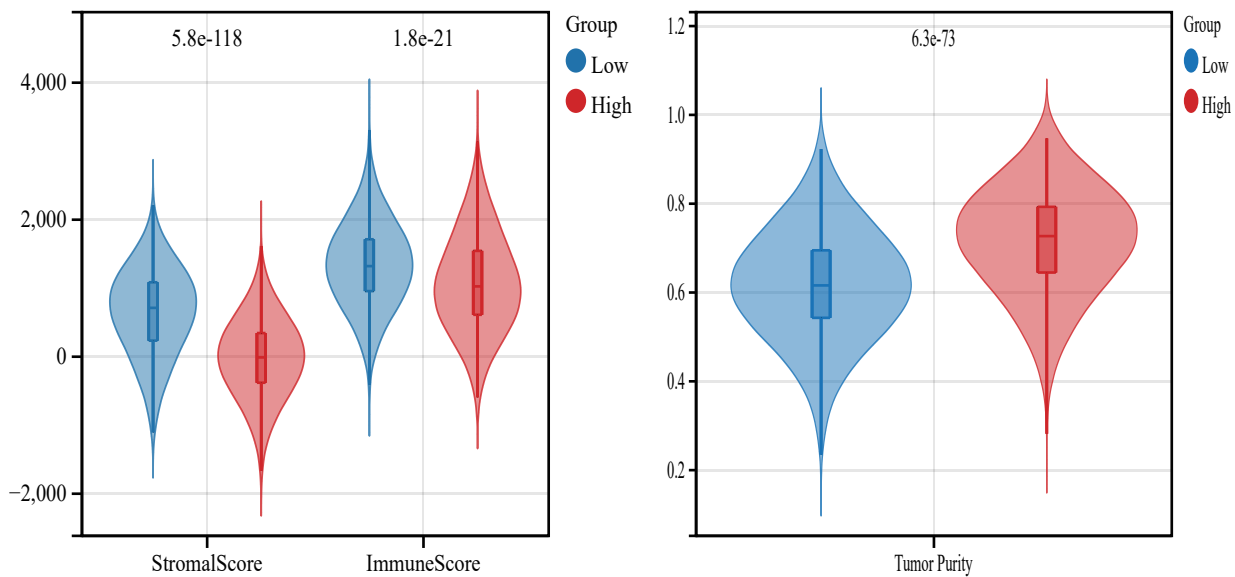**B**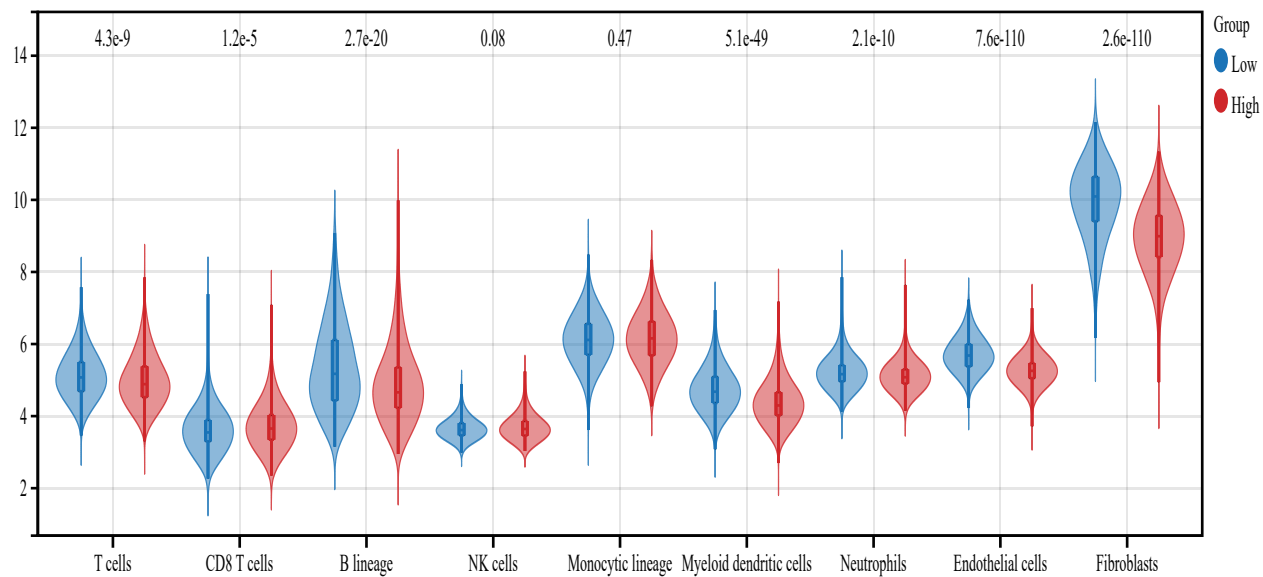

Supplement: Supplementary Figure 3 — Tumor microenvironment features underlying the DDR pathway signature. (A) Violin plots show the different distribution of stromal score, immune score, and tumor purity between the high and low DDR signature score groups. (B) Violin plots show the different distribution of stromal and immune cells infiltration levels between the high and low DDR signature score groups. [file Image_3.pdf]

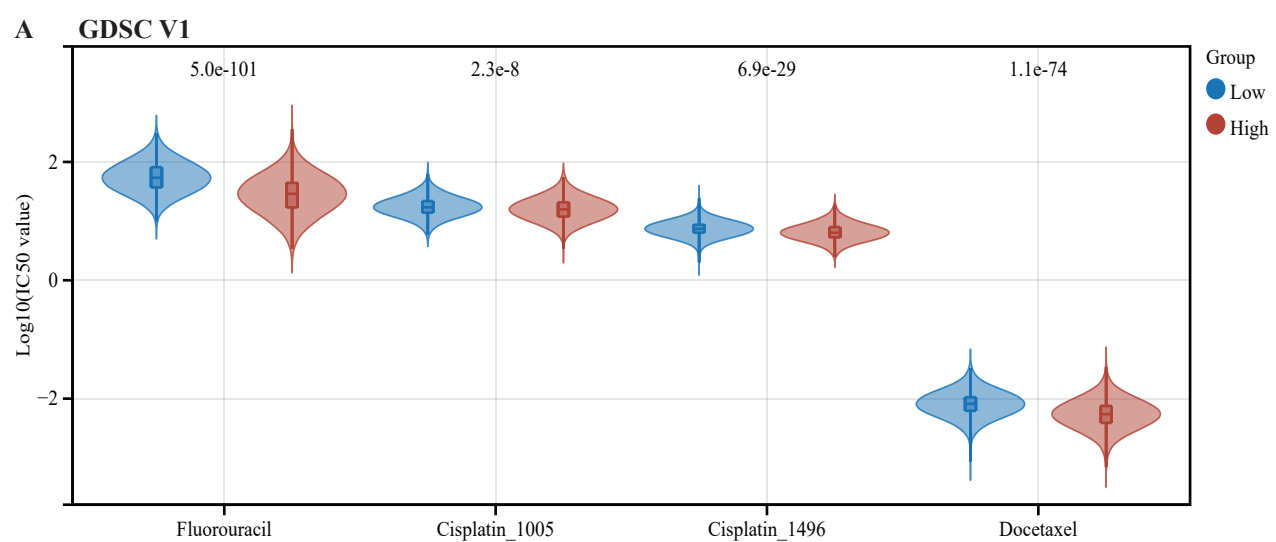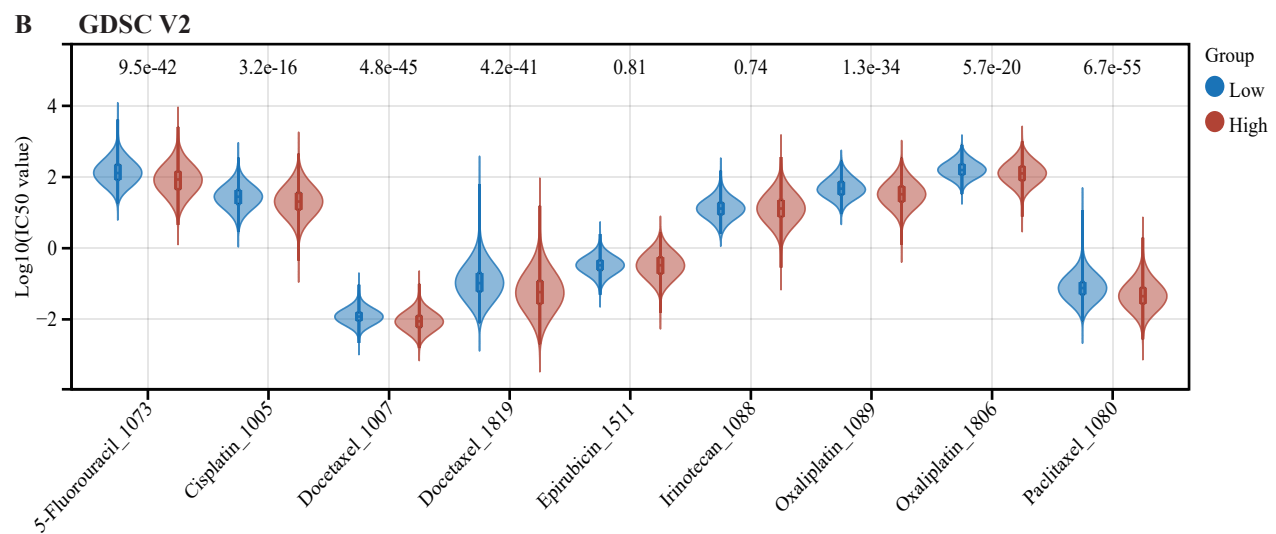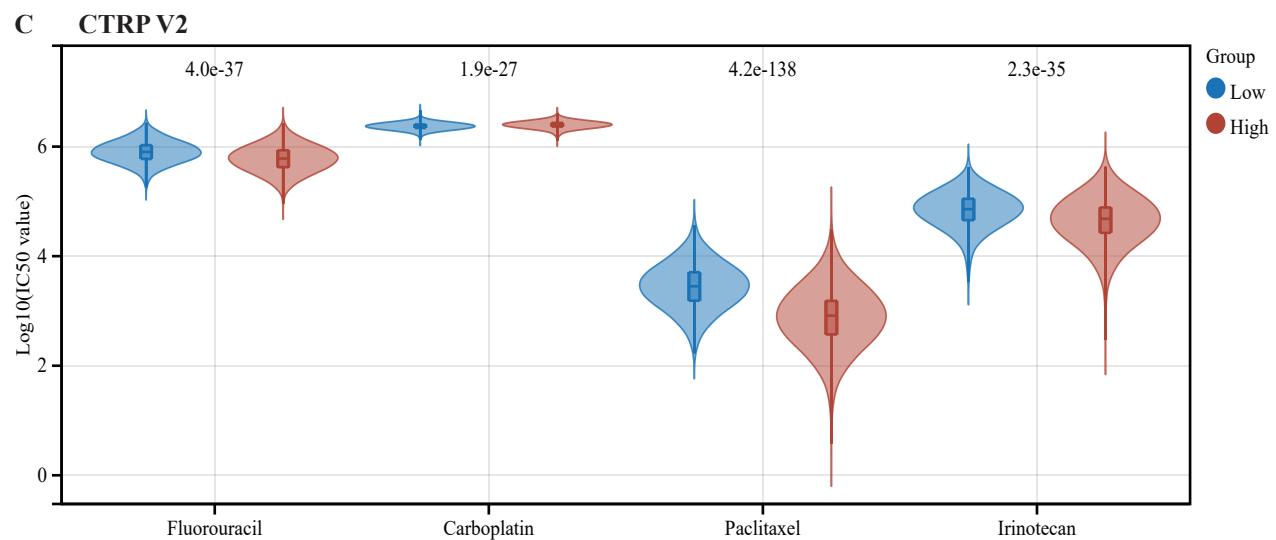

Supplement: Supplementary Figure 4 — Potential response to chemotherapy. Violin plots show the different distribution of the IC50 values for the selected chemotherapeutic agents in the high and low DDR signature score groups based on (A) GDSC V1, (B) GDSC V2, and (C) CTRP V2 database. [file Image_4.pdf]
