# Supplementary material for: Patient-Level DNA Damage Repair Pathway Profiles and Anti-Tumor Immunity for Gastric Cancer
Source: Front Immunol. 2022 Jan 10;12:806324. doi: 10.3389/fimmu.2021.806324 (PMC8785952; doi:10.3389/fimmu.2021.806324)
Supplement: Supplementary Figure 5 — The research workflow of this study. [file Image_5.pdf]

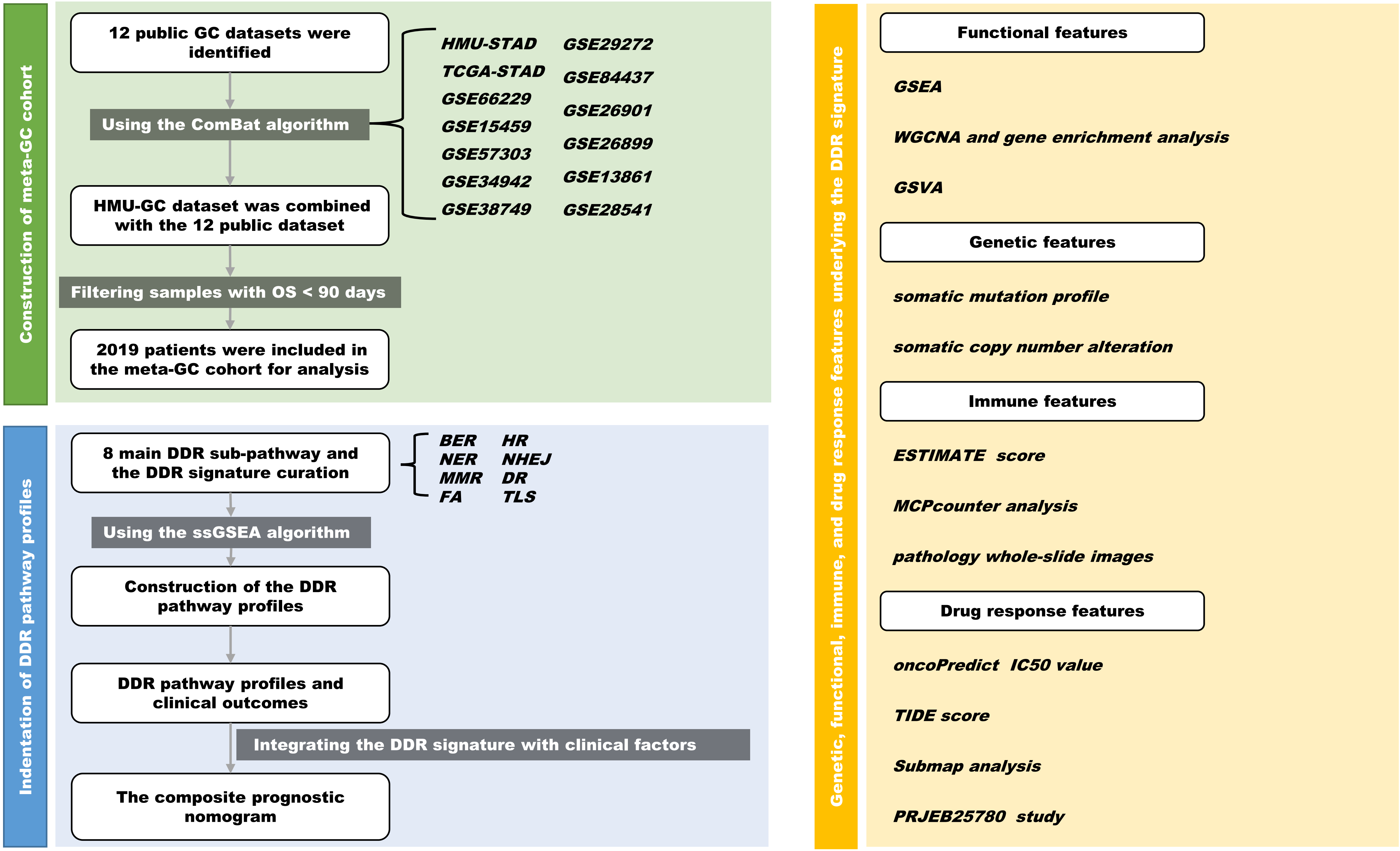
Construction of meta-GC cohort
12 public GC datasets were identified
Using the ComBat algorithm
HMU-GC dataset was combined with the 12 public dataset
Filtering samples with OS < 90 days
2019 patients were included in the meta-GC cohort for analysis
HMU-STAD
TCGA-STAD
GSE66229
GSE15459
GSE57303
GSE34942
GSE38749
GSE29272
GSE84437
GSE26901
GSE26899
GSE13861
GSE28541
Indentation of DDR pathway profiles
8 main DDR sub-pathway and the DDR signature curation
BER
NER
MMR
FA
HR
NHEJ
DR
TLS
Using the ssGSEA algorithm
Construction of the DDR pathway profiles
DDR pathway profiles and clinical outcomes
Integrating the DDR signature with clinical factors
The composite prognostic nomogram
Genetic, functional, immune, and drug response features underlying the DDR signature
Functional features
GSEA
WGCNA and gene enrichment analysis
GSVA
Genetic features
somatic mutation profile
somatic copy number alteration
Immune features
ESTIMATE score
MCPcounter analysis
pathology whole-slide images
Drug response features
oncoPredict IC50 value
TIDE score
Submap analysis
PRJEB25780 study
