# Supplementary material for: Patient-Level DNA Damage Repair Pathway Profiles and Anti-Tumor Immunity for Gastric Cancer
Source: Front Immunol. 2022 Jan 10;12:806324. doi: 10.3389/fimmu.2021.806324 (PMC8785952; doi:10.3389/fimmu.2021.806324)
Supplement: Supplementary Table 1 — The basic information of samples in the 12 public datasets. [file Table_1.docx]

**Table S1.** The basic information of samples in the 12 public datasets.

| **Dataset** | **Sample size** | **Platform** | **Data type** |
| --- | --- | --- | --- |
| TCGA-STAD | 375 | High-throughput sequencing | mRNA data, CNV data, Mutation data |
| ACRG | 300 | Microarray | mRNA data, CNV data |
| GSE15459 | 192 | Microarray | mRNA data |
| GSE57303 | 70 | Microarray | mRNA data |
| GSE34942 | 56 | Microarray | mRNA data |
| GSE38749 | 15 | Microarray | mRNA data |
| GSE29272 | 134 | Microarray | mRNA data |
| GSE84437 | 433 | Microarray | mRNA data |
| GSE26901 | 109 | Microarray | mRNA data |
| GSE26899 | 96 | Microarray | mRNA data |
| GSE13861 | 65 | Microarray | mRNA data |
| GSE28541 | 40 | Microarray | mRNA data |
