# Supplementary material for: Patient-Level DNA Damage Repair Pathway Profiles and Anti-Tumor Immunity for Gastric Cancer
Source: Front Immunol. 2022 Jan 10;12:806324. doi: 10.3389/fimmu.2021.806324 (PMC8785952; doi:10.3389/fimmu.2021.806324)
Supplement: Supplementary Table 3 — Gene mutation rates between the high and low DDR signature score groups in the TCGA cohort. [file Table_3.docx]

Table S3. INDEPENDENT TEST BETWEEN SUBTYPE AND MUTATION

| Gene (Mutated) | TMB | CS1 | CS2 | pvalue | padj |
| --- | --- | --- | --- | --- | --- |
| PTEN | 26 ( 7%) | 6 (3.7%) | 20 (9.6%) | 3.86e-02 | 8.21e-02 |
| MYOF | 21 ( 6%) | 5 (3.1%) | 16 (7.7%) | 7.03e-02 | 1.17e-01 |
| DCHS1 | 29 ( 8%) | 4 ( 2.5%) | 25 (12.0%) | 6.66e-04 | 1.08e-02 |
| TENM4 | 36 (10%) | 6 ( 3.7%) | 30 (14.4%) | 5.99e-04 | 1.08e-02 |
| HECTD4 | 28 ( 8%) | 7 ( 4.3%) | 21 (10.1%) | 4.67e-02 | 9.50e-02 |
| KRAS | 32 ( 9%) | 9 ( 5.6%) | 23 (11.1%) | 6.51e-02 | 1.14e-01 |
| ARID2 | 28 ( 8%) | 9 (5.6%) | 19 (9.1%) | 2.37e-01 | 3.08e-01 |
| MYH7 | 31 ( 8%) | 7 ( 4.3%) | 24 (11.5%) | 1.38e-02 | 4.47e-02 |
| NPAP1 | 29 ( 8%) | 5 ( 3.1%) | 24 (11.5%) | 2.92e-03 | 2.00e-02 |
| SRCAP | 30 ( 8%) | 6 ( 3.7%) | 24 (11.5%) | 6.66e-03 | 3.24e-02 |
| GRIN2A | 21 ( 6%) | 6 (3.7%) | 15 (7.2%) | 1.78e-01 | 2.40e-01 |
| MYH13 | 19 ( 5%) | 5 (3.1%) | 14 (6.7%) | 1.55e-01 | 2.17e-01 |
| NF1 | 28 ( 8%) | 7 ( 4.3%) | 21 (10.1%) | 4.67e-02 | 9.50e-02 |
| KIAA0195 | 20 ( 5%) | 1 (0.6%) | 19 (9.1%) | 1.33e-04 | 6.72e-03 |
| TP53 | 181 (49%) | 58 (35.8%) | 123 (59.1%) | 1.01e-05 | 3.53e-03 |
| FASN | 19 ( 5%) | 4 (2.5%) | 15 (7.2%) | 5.56e-02 | 1.04e-01 |
| TSHZ3 | 24 ( 6%) | 10 (6.2%) | 14 (6.7%) | 1.00e+00 | 1.00e+00 |
| ZNF667 | 19 ( 5%) | 8 (4.9%) | 11 (5.3%) | 1.00e+00 | 1.00e+00 |
| F5 | 22 ( 6%) | 7 (4.3%) | 15 (7.2%) | 2.75e-01 | 3.37e-01 |
| TNR | 20 ( 5%) | 8 (4.9%) | 12 (5.8%) | 8.19e-01 | 8.57e-01 |
| PAPPA2 | 31 ( 8%) | 8 ( 4.9%) | 23 (11.1%) | 3.83e-02 | 8.21e-02 |
| RYR2 | 67 (18%) | 20 (12.3%) | 47 (22.6%) | 1.39e-02 | 4.47e-02 |
| DIDO1 | 33 ( 9%) | 4 ( 2.5%) | 29 (13.9%) | 7.32e-05 | 6.72e-03 |
| ADAMTS5 | 26 ( 7%) | 5 ( 3.1%) | 21 (10.1%) | 1.25e-02 | 4.47e-02 |
| TTC3 | 19 ( 5%) | 5 (3.1%) | 14 (6.7%) | 1.55e-01 | 2.17e-01 |
| STXBP5L | 20 ( 5%) | 6 (3.7%) | 14 (6.7%) | 2.50e-01 | 3.16e-01 |
| POLQ | 29 ( 8%) | 8 ( 4.9%) | 21 (10.1%) | 7.99e-02 | 1.30e-01 |
| GOLGB1 | 27 ( 7%) | 5 ( 3.1%) | 22 (10.6%) | 7.77e-03 | 3.49e-02 |
| ANK2 | 36 (10%) | 8 ( 4.9%) | 28 (13.5%) | 7.36e-03 | 3.48e-02 |
| FBXW7 | 28 ( 8%) | 8 (4.9%) | 20 (9.6%) | 1.13e-01 | 1.70e-01 |
| TENM2 | 21 ( 6%) | 5 (3.1%) | 16 (7.7%) | 7.03e-02 | 1.17e-01 |
| SLIT3 | 21 ( 6%) | 0 ( 0.0%) | 21 (10.1%) | 3.93e-06 | 2.75e-03 |
| MLLT4 | 21 ( 6%) | 3 (1.9%) | 18 (8.7%) | 5.54e-03 | 2.83e-02 |
| BAI3 | 43 (12%) | 13 ( 8.0%) | 30 (14.4%) | 7.16e-02 | 1.18e-01 |
| ELMO1 | 20 ( 5%) | 6 (3.7%) | 14 (6.7%) | 2.50e-01 | 3.16e-01 |
| CSMD3 | 96 (26%) | 25 (15.4%) | 71 (34.1%) | 4.40e-05 | 6.72e-03 |
| C9ORF84 | 20 ( 5%) | 5 (3.1%) | 15 (7.2%) | 1.05e-01 | 1.62e-01 |
| FREM1 | 24 ( 6%) | 5 (3.1%) | 19 (9.1%) | 1.98e-02 | 5.50e-02 |
| PRUNE2 | 22 ( 6%) | 9 (5.6%) | 13 (6.2%) | 8.28e-01 | 8.60e-01 |
| CTNNA3 | 22 ( 6%) | 7 (4.3%) | 15 (7.2%) | 2.75e-01 | 3.37e-01 |
| NAV3 | 43 (12%) | 17 (10.5%) | 26 (12.5%) | 6.25e-01 | 6.73e-01 |
| DCLK1 | 38 (10%) | 11 ( 6.8%) | 27 (13.0%) | 5.83e-02 | 1.05e-01 |
| FLRT2 | 25 ( 7%) | 10 (6.2%) | 15 (7.2%) | 8.35e-01 | 8.60e-01 |
| ZNF646 | 22 ( 6%) | 5 (3.1%) | 17 (8.2%) | 4.65e-02 | 9.50e-02 |
| SALL1 | 25 ( 7%) | 10 (6.2%) | 15 (7.2%) | 8.35e-01 | 8.60e-01 |
| CDH8 | 31 ( 8%) | 12 (7.4%) | 19 (9.1%) | 5.77e-01 | 6.24e-01 |
| CDH1 | 33 ( 9%) | 23 (14.2%) | 10 ( 4.8%) | 2.76e-03 | 2.00e-02 |
| HYDIN | 35 (10%) | 9 ( 5.6%) | 26 (12.5%) | 3.07e-02 | 7.33e-02 |
| SCN4A | 21 ( 6%) | 6 (3.7%) | 15 (7.2%) | 1.78e-01 | 2.40e-01 |
| TCEB3B | 24 ( 6%) | 5 (3.1%) | 19 (9.1%) | 1.98e-02 | 5.50e-02 |
| KMT2B | 33 ( 9%) | 8 ( 4.9%) | 25 (12.0%) | 2.61e-02 | 6.48e-02 |
| RANBP2 | 28 ( 8%) | 7 ( 4.3%) | 21 (10.1%) | 4.67e-02 | 9.50e-02 |
| ACVR2A | 41 (11%) | 12 ( 7.4%) | 29 (13.9%) | 6.53e-02 | 1.14e-01 |
| PXDN | 43 (12%) | 15 ( 9.3%) | 28 (13.5%) | 2.53e-01 | 3.19e-01 |
| FN1 | 22 ( 6%) | 4 (2.5%) | 18 (8.7%) | 1.40e-02 | 4.47e-02 |
| SCN5A | 30 ( 8%) | 9 ( 5.6%) | 21 (10.1%) | 1.27e-01 | 1.88e-01 |
| PCDH10 | 42 (11%) | 11 ( 6.8%) | 31 (14.9%) | 1.99e-02 | 5.51e-02 |
| GRID2 | 21 ( 6%) | 6 (3.7%) | 15 (7.2%) | 1.78e-01 | 2.40e-01 |
| NSD1 | 20 ( 5%) | 4 (2.5%) | 16 (7.7%) | 3.55e-02 | 7.74e-02 |
| HCN1 | 25 ( 7%) | 7 (4.3%) | 18 (8.7%) | 1.43e-01 | 2.07e-01 |
| ASCC3 | 29 ( 8%) | 6 ( 3.7%) | 23 (11.1%) | 1.05e-02 | 4.06e-02 |
| AKAP12 | 19 ( 5%) | 4 (2.5%) | 15 (7.2%) | 5.56e-02 | 1.04e-01 |
| COL12A1 | 53 (14%) | 16 ( 9.9%) | 37 (17.8%) | 3.61e-02 | 7.82e-02 |
| POM121L12 | 19 ( 5%) | 3 (1.9%) | 16 (7.7%) | 1.56e-02 | 4.61e-02 |
| MAGI2 | 23 ( 6%) | 2 ( 1.2%) | 21 (10.1%) | 3.13e-04 | 7.56e-03 |
| RIMS2 | 42 (11%) | 12 ( 7.4%) | 30 (14.4%) | 4.65e-02 | 9.50e-02 |
| DLC1 | 28 ( 8%) | 5 ( 3.1%) | 23 (11.1%) | 4.78e-03 | 2.66e-02 |
| FAM135B | 30 ( 8%) | 11 (6.8%) | 19 (9.1%) | 4.49e-01 | 5.00e-01 |
| KCNB2 | 26 ( 7%) | 8 (4.9%) | 18 (8.7%) | 2.19e-01 | 2.85e-01 |
| ZFHX4 | 65 (18%) | 16 ( 9.9%) | 49 (23.6%) | 5.52e-04 | 1.08e-02 |
| COL15A1 | 22 ( 6%) | 6 (3.7%) | 16 (7.7%) | 1.24e-01 | 1.85e-01 |
| BNC2 | 21 ( 6%) | 5 (3.1%) | 16 (7.7%) | 7.03e-02 | 1.17e-01 |
| SORCS3 | 20 ( 5%) | 8 (4.9%) | 12 (5.8%) | 8.19e-01 | 8.57e-01 |
| SVIL | 22 ( 6%) | 4 (2.5%) | 18 (8.7%) | 1.40e-02 | 4.47e-02 |
| DIP2C | 21 ( 6%) | 2 (1.2%) | 19 (9.1%) | 1.01e-03 | 1.44e-02 |
| GRID1 | 20 ( 5%) | 6 (3.7%) | 14 (6.7%) | 2.50e-01 | 3.16e-01 |
| BTAF1 | 19 ( 5%) | 6 (3.7%) | 13 (6.2%) | 3.45e-01 | 4.07e-01 |
| MUC6 | 37 (10%) | 11 ( 6.8%) | 26 (12.5%) | 8.12e-02 | 1.32e-01 |
| KMT2A | 37 (10%) | 9 ( 5.6%) | 28 (13.5%) | 1.39e-02 | 4.47e-02 |
| TECTA | 34 ( 9%) | 11 ( 6.8%) | 23 (11.1%) | 2.04e-01 | 2.68e-01 |
| SORL1 | 21 ( 6%) | 4 (2.5%) | 17 (8.2%) | 2.24e-02 | 5.76e-02 |
| ABCC8 | 19 ( 5%) | 5 (3.1%) | 14 (6.7%) | 1.55e-01 | 2.17e-01 |
| DCDC1 | 28 ( 8%) | 8 (4.9%) | 20 (9.6%) | 1.13e-01 | 1.70e-01 |
| PTPRJ | 23 ( 6%) | 4 (2.5%) | 19 (9.1%) | 8.69e-03 | 3.69e-02 |
| LRP5 | 19 ( 5%) | 4 (2.5%) | 15 (7.2%) | 5.56e-02 | 1.04e-01 |
| SCUBE2 | 21 ( 6%) | 7 (4.3%) | 14 (6.7%) | 3.71e-01 | 4.27e-01 |
| BTBD11 | 25 ( 7%) | 10 (6.2%) | 15 (7.2%) | 8.35e-01 | 8.60e-01 |
| NCOR2 | 27 ( 7%) | 7 (4.3%) | 20 (9.6%) | 6.87e-02 | 1.17e-01 |
| EP400 | 29 ( 8%) | 4 ( 2.5%) | 25 (12.0%) | 6.66e-04 | 1.08e-02 |
| ABCC9 | 35 (10%) | 12 ( 7.4%) | 23 (11.1%) | 2.84e-01 | 3.47e-01 |
| CHD4 | 26 ( 7%) | 6 (3.7%) | 20 (9.6%) | 3.86e-02 | 8.21e-02 |
| PPFIA2 | 21 ( 6%) | 6 (3.7%) | 15 (7.2%) | 1.78e-01 | 2.40e-01 |
| LRRIQ1 | 24 ( 6%) | 7 (4.3%) | 17 (8.2%) | 2.01e-01 | 2.65e-01 |
| A2M | 22 ( 6%) | 10 (6.2%) | 12 (5.8%) | 1.00e+00 | 1.00e+00 |
| NALCN | 37 (10%) | 14 ( 8.6%) | 23 (11.1%) | 4.88e-01 | 5.36e-01 |
| MYO16 | 24 ( 6%) | 8 (4.9%) | 16 (7.7%) | 3.95e-01 | 4.51e-01 |
| PCDH17 | 39 (10%) | 9 ( 5.6%) | 30 (14.4%) | 6.10e-03 | 3.01e-02 |
| PCDH9 | 24 ( 6%) | 6 (3.7%) | 18 (8.7%) | 5.87e-02 | 1.05e-01 |
| SLITRK5 | 37 (10%) | 8 ( 4.9%) | 29 (13.9%) | 4.74e-03 | 2.66e-02 |
| SIPA1L1 | 28 ( 8%) | 6 ( 3.7%) | 22 (10.6%) | 1.64e-02 | 4.70e-02 |
| ATP10A | 37 (10%) | 5 ( 3.1%) | 32 (15.4%) | 7.63e-05 | 6.72e-03 |
| TJP1 | 22 ( 6%) | 4 (2.5%) | 18 (8.7%) | 1.40e-02 | 4.47e-02 |
| MGA | 29 ( 8%) | 5 ( 3.1%) | 24 (11.5%) | 2.92e-03 | 2.00e-02 |
| TP53BP1 | 19 ( 5%) | 3 (1.9%) | 16 (7.7%) | 1.56e-02 | 4.61e-02 |
| UNC13C | 33 ( 9%) | 5 ( 3.1%) | 28 (13.5%) | 3.84e-04 | 8.96e-03 |
| TLN2 | 25 ( 7%) | 8 (4.9%) | 17 (8.2%) | 2.97e-01 | 3.60e-01 |
| HERC1 | 34 ( 9%) | 5 ( 3.1%) | 29 (13.9%) | 2.29e-04 | 6.72e-03 |
| PEAK1 | 19 ( 5%) | 4 (2.5%) | 15 (7.2%) | 5.56e-02 | 1.04e-01 |
| MYH11 | 25 ( 7%) | 8 (4.9%) | 17 (8.2%) | 2.97e-01 | 3.60e-01 |
| GTF3C1 | 28 ( 8%) | 5 ( 3.1%) | 23 (11.1%) | 4.78e-03 | 2.66e-02 |
| SLX4 | 21 ( 6%) | 6 (3.7%) | 15 (7.2%) | 1.78e-01 | 2.40e-01 |
| CREBBP | 30 ( 8%) | 11 (6.8%) | 19 (9.1%) | 4.49e-01 | 5.00e-01 |
| CDH11 | 31 ( 8%) | 11 (6.8%) | 20 (9.6%) | 3.52e-01 | 4.13e-01 |
| CTCF | 21 ( 6%) | 5 (3.1%) | 16 (7.7%) | 7.03e-02 | 1.17e-01 |
| ANKRD11 | 32 ( 9%) | 5 ( 3.1%) | 27 (13.0%) | 6.43e-04 | 1.08e-02 |
| MYH4 | 20 ( 5%) | 3 (1.9%) | 17 (8.2%) | 9.33e-03 | 3.69e-02 |
| WNK4 | 19 ( 5%) | 1 (0.6%) | 18 (8.7%) | 2.40e-04 | 6.72e-03 |
| GPATCH8 | 19 ( 5%) | 1 (0.6%) | 18 (8.7%) | 2.40e-04 | 6.72e-03 |
| CACNA1G | 19 ( 5%) | 5 (3.1%) | 14 (6.7%) | 1.55e-01 | 2.17e-01 |
| KIF2B | 27 ( 7%) | 9 (5.6%) | 18 (8.7%) | 3.16e-01 | 3.81e-01 |
| EVPL | 19 ( 5%) | 4 (2.5%) | 15 (7.2%) | 5.56e-02 | 1.04e-01 |
| PTPRM | 28 ( 8%) | 7 ( 4.3%) | 21 (10.1%) | 4.67e-02 | 9.50e-02 |
| SIPA1L3 | 24 ( 6%) | 9 (5.6%) | 15 (7.2%) | 6.71e-01 | 7.14e-01 |
| SPTBN4 | 25 ( 7%) | 6 (3.7%) | 19 (9.1%) | 5.83e-02 | 1.05e-01 |
| LRRC4B | 19 ( 5%) | 4 (2.5%) | 15 (7.2%) | 5.56e-02 | 1.04e-01 |
| C3 | 23 ( 6%) | 4 (2.5%) | 19 (9.1%) | 8.69e-03 | 3.69e-02 |
| ADAMTS10 | 21 ( 6%) | 4 (2.5%) | 17 (8.2%) | 2.24e-02 | 5.76e-02 |
| SPAG17 | 20 ( 5%) | 3 (1.9%) | 17 (8.2%) | 9.33e-03 | 3.69e-02 |
| FCRL3 | 19 ( 5%) | 8 (4.9%) | 11 (5.3%) | 1.00e+00 | 1.00e+00 |
| SLC9C2 | 21 ( 6%) | 4 (2.5%) | 17 (8.2%) | 2.24e-02 | 5.76e-02 |
| CACNA1E | 41 (11%) | 10 ( 6.2%) | 31 (14.9%) | 7.73e-03 | 3.49e-02 |
| CFH | 24 ( 6%) | 7 (4.3%) | 17 (8.2%) | 2.01e-01 | 2.65e-01 |
| KCNH1 | 19 ( 5%) | 4 (2.5%) | 15 (7.2%) | 5.56e-02 | 1.04e-01 |
| CENPF | 26 ( 7%) | 5 ( 3.1%) | 21 (10.1%) | 1.25e-02 | 4.47e-02 |
| KIF26B | 22 ( 6%) | 5 (3.1%) | 17 (8.2%) | 4.65e-02 | 9.50e-02 |
| ARID1A | 89 (24%) | 26 (16.0%) | 63 (30.3%) | 1.45e-03 | 1.79e-02 |
| HIVEP3 | 28 ( 8%) | 3 ( 1.9%) | 25 (12.0%) | 2.19e-04 | 6.72e-03 |
| RERE | 25 ( 7%) | 3 ( 1.9%) | 22 (10.6%) | 6.56e-04 | 1.08e-02 |
| ZNFX1 | 19 ( 5%) | 4 (2.5%) | 15 (7.2%) | 5.56e-02 | 1.04e-01 |
| NFATC2 | 19 ( 5%) | 4 (2.5%) | 15 (7.2%) | 5.56e-02 | 1.04e-01 |
| TMPRSS15 | 19 ( 5%) | 10 (6.2%) | 9 (4.3%) | 4.81e-01 | 5.34e-01 |
| TIAM1 | 28 ( 8%) | 8 (4.9%) | 20 (9.6%) | 1.13e-01 | 1.70e-01 |
| SCAF4 | 24 ( 6%) | 4 (2.5%) | 20 (9.6%) | 5.35e-03 | 2.83e-02 |
| BRWD1 | 22 ( 6%) | 7 (4.3%) | 15 (7.2%) | 2.75e-01 | 3.37e-01 |
| COL6A2 | 20 ( 5%) | 4 (2.5%) | 16 (7.7%) | 3.55e-02 | 7.74e-02 |
| TRIOBP | 28 ( 8%) | 9 (5.6%) | 19 (9.1%) | 2.37e-01 | 3.08e-01 |
| TUBGCP6 | 21 ( 6%) | 5 (3.1%) | 16 (7.7%) | 7.03e-02 | 1.17e-01 |
| RGPD4 | 19 ( 5%) | 1 (0.6%) | 18 (8.7%) | 2.40e-04 | 6.72e-03 |
| CNTNAP5 | 37 (10%) | 9 ( 5.6%) | 28 (13.5%) | 1.39e-02 | 4.47e-02 |
| TPO | 22 ( 6%) | 6 (3.7%) | 16 (7.7%) | 1.24e-01 | 1.85e-01 |
| RIF1 | 24 ( 6%) | 4 (2.5%) | 20 (9.6%) | 5.35e-03 | 2.83e-02 |
| ZNF804A | 26 ( 7%) | 7 (4.3%) | 19 (9.1%) | 9.98e-02 | 1.58e-01 |
| FSIP2 | 28 ( 8%) | 6 ( 3.7%) | 22 (10.6%) | 1.64e-02 | 4.70e-02 |
| ERBB4 | 43 (12%) | 16 ( 9.9%) | 27 (13.0%) | 4.15e-01 | 4.66e-01 |
| TNS1 | 21 ( 6%) | 4 (2.5%) | 17 (8.2%) | 2.24e-02 | 5.76e-02 |
| SPEG | 27 ( 7%) | 6 ( 3.7%) | 21 (10.1%) | 2.53e-02 | 6.35e-02 |
| SPHKAP | 22 ( 6%) | 7 (4.3%) | 15 (7.2%) | 2.75e-01 | 3.37e-01 |
| HDAC4 | 20 ( 5%) | 2 (1.2%) | 18 (8.7%) | 1.82e-03 | 1.79e-02 |
| HDLBP | 23 ( 6%) | 3 (1.9%) | 20 (9.6%) | 1.92e-03 | 1.79e-02 |
| OTOF | 19 ( 5%) | 6 (3.7%) | 13 (6.2%) | 3.45e-01 | 4.07e-01 |
| CAD | 20 ( 5%) | 3 (1.9%) | 17 (8.2%) | 9.33e-03 | 3.69e-02 |
| SPTBN1 | 20 ( 5%) | 4 (2.5%) | 16 (7.7%) | 3.55e-02 | 7.74e-02 |
| CCDC88A | 21 ( 6%) | 6 (3.7%) | 15 (7.2%) | 1.78e-01 | 2.40e-01 |
| BCL11A | 20 ( 5%) | 5 (3.1%) | 15 (7.2%) | 1.05e-01 | 1.62e-01 |
| USP34 | 25 ( 7%) | 6 (3.7%) | 19 (9.1%) | 5.83e-02 | 1.05e-01 |
| DYSF | 29 ( 8%) | 8 ( 4.9%) | 21 (10.1%) | 7.99e-02 | 1.30e-01 |
| NUP210 | 21 ( 6%) | 4 (2.5%) | 17 (8.2%) | 2.24e-02 | 5.76e-02 |
| EPHB1 | 22 ( 6%) | 9 (5.6%) | 13 (6.2%) | 8.28e-01 | 8.60e-01 |
| MED12L | 32 ( 9%) | 6 ( 3.7%) | 26 (12.5%) | 2.64e-03 | 2.00e-02 |
| IGSF10 | 36 (10%) | 8 ( 4.9%) | 28 (13.5%) | 7.36e-03 | 3.48e-02 |
| PIK3CA | 61 (16%) | 13 ( 8.0%) | 48 (23.1%) | 1.11e-04 | 6.72e-03 |
| CNTN4 | 21 ( 6%) | 7 (4.3%) | 14 (6.7%) | 3.71e-01 | 4.27e-01 |
| GOLGA4 | 19 ( 5%) | 5 (3.1%) | 14 (6.7%) | 1.55e-01 | 2.17e-01 |
| CTNNB1 | 21 ( 6%) | 6 (3.7%) | 15 (7.2%) | 1.78e-01 | 2.40e-01 |
| PLXNB1 | 19 ( 5%) | 2 (1.2%) | 17 (8.2%) | 3.24e-03 | 2.00e-02 |
| CELSR3 | 36 (10%) | 6 ( 3.7%) | 30 (14.4%) | 5.99e-04 | 1.08e-02 |
| PBRM1 | 20 ( 5%) | 3 (1.9%) | 17 (8.2%) | 9.33e-03 | 3.69e-02 |
| CACNA1D | 23 ( 6%) | 3 (1.9%) | 20 (9.6%) | 1.92e-03 | 1.79e-02 |
| KIAA1109 | 34 ( 9%) | 13 ( 8.0%) | 21 (10.1%) | 5.88e-01 | 6.34e-01 |
| DCHS2 | 37 (10%) | 8 ( 4.9%) | 29 (13.9%) | 4.74e-03 | 2.66e-02 |
| TLL1 | 20 ( 5%) | 9 (5.6%) | 11 (5.3%) | 1.00e+00 | 1.00e+00 |
| TENM3 | 37 (10%) | 12 ( 7.4%) | 25 (12.0%) | 1.64e-01 | 2.29e-01 |
| HTT | 23 ( 6%) | 6 (3.7%) | 17 (8.2%) | 8.60e-02 | 1.37e-01 |
| RGS12 | 32 ( 9%) | 7 ( 4.3%) | 25 (12.0%) | 9.00e-03 | 3.69e-02 |
| ATP10D | 22 ( 6%) | 4 (2.5%) | 18 (8.7%) | 1.40e-02 | 4.47e-02 |
| SLCO6A1 | 19 ( 5%) | 3 (1.9%) | 16 (7.7%) | 1.56e-02 | 4.61e-02 |
| PCDHA1 | 26 ( 7%) | 5 ( 3.1%) | 21 (10.1%) | 1.25e-02 | 4.47e-02 |
| PCDHA11 | 30 ( 8%) | 5 ( 3.1%) | 25 (12.0%) | 1.77e-03 | 1.79e-02 |
| PCDHB2 | 22 ( 6%) | 9 (5.6%) | 13 (6.2%) | 8.28e-01 | 8.60e-01 |
| PCDHB5 | 22 ( 6%) | 7 (4.3%) | 15 (7.2%) | 2.75e-01 | 3.37e-01 |
| PCDHB6 | 22 ( 6%) | 4 (2.5%) | 18 (8.7%) | 1.40e-02 | 4.47e-02 |
| PCDHB7 | 22 ( 6%) | 3 (1.9%) | 19 (9.1%) | 3.27e-03 | 2.00e-02 |
| PCDHB8 | 20 ( 5%) | 4 (2.5%) | 16 (7.7%) | 3.55e-02 | 7.74e-02 |
| PCDHB12 | 20 ( 5%) | 2 (1.2%) | 18 (8.7%) | 1.82e-03 | 1.79e-02 |
| CDHR2 | 20 ( 5%) | 4 (2.5%) | 16 (7.7%) | 3.55e-02 | 7.74e-02 |
| PRDM9 | 21 ( 6%) | 9 (5.6%) | 12 (5.8%) | 1.00e+00 | 1.00e+00 |
| PDZD2 | 34 ( 9%) | 7 ( 4.3%) | 27 (13.0%) | 5.79e-03 | 2.92e-02 |
| VCAN | 30 ( 8%) | 5 ( 3.1%) | 25 (12.0%) | 1.77e-03 | 1.79e-02 |
| REV3L | 37 (10%) | 8 ( 4.9%) | 29 (13.9%) | 4.74e-03 | 2.66e-02 |
| LAMA4 | 24 ( 6%) | 7 (4.3%) | 17 (8.2%) | 2.01e-01 | 2.65e-01 |
| GRM1 | 35 (10%) | 8 ( 4.9%) | 27 (13.0%) | 1.13e-02 | 4.32e-02 |
| TIAM2 | 20 ( 5%) | 6 (3.7%) | 14 (6.7%) | 2.50e-01 | 3.16e-01 |
| PRRC2A | 19 ( 5%) | 7 (4.3%) | 12 (5.8%) | 6.38e-01 | 6.84e-01 |
| TNXB | 32 ( 9%) | 9 ( 5.6%) | 23 (11.1%) | 6.51e-02 | 1.14e-01 |
| CUX1 | 20 ( 5%) | 2 (1.2%) | 18 (8.7%) | 1.82e-03 | 1.79e-02 |
| PIK3CG | 20 ( 5%) | 1 (0.6%) | 19 (9.1%) | 1.33e-04 | 6.72e-03 |
| LAMB4 | 19 ( 5%) | 4 (2.5%) | 15 (7.2%) | 5.56e-02 | 1.04e-01 |
| THSD7A | 22 ( 6%) | 6 (3.7%) | 16 (7.7%) | 1.24e-01 | 1.85e-01 |
| PTPRZ1 | 23 ( 6%) | 4 (2.5%) | 19 (9.1%) | 8.69e-03 | 3.69e-02 |
| GRM8 | 21 ( 6%) | 4 (2.5%) | 17 (8.2%) | 2.24e-02 | 5.76e-02 |
| FLNC | 29 ( 8%) | 3 ( 1.9%) | 26 (12.5%) | 1.21e-04 | 6.72e-03 |
| TNS3 | 21 ( 6%) | 3 (1.9%) | 18 (8.7%) | 5.54e-03 | 2.83e-02 |
| ABCB1 | 26 ( 7%) | 11 (6.8%) | 15 (7.2%) | 1.00e+00 | 1.00e+00 |
| TRRAP | 41 (11%) | 10 ( 6.2%) | 31 (14.9%) | 7.73e-03 | 3.49e-02 |
| RGS22 | 20 ( 5%) | 4 (2.5%) | 16 (7.7%) | 3.55e-02 | 7.74e-02 |
| UBR5 | 36 (10%) | 12 ( 7.4%) | 24 (11.5%) | 2.17e-01 | 2.84e-01 |
| TRPS1 | 35 (10%) | 14 ( 8.6%) | 21 (10.1%) | 7.22e-01 | 7.62e-01 |
| COL22A1 | 28 ( 8%) | 6 ( 3.7%) | 22 (10.6%) | 1.64e-02 | 4.70e-02 |
| ANK1 | 28 ( 8%) | 8 (4.9%) | 20 (9.6%) | 1.13e-01 | 1.70e-01 |
| PRKDC | 34 ( 9%) | 15 (9.3%) | 19 (9.1%) | 1.00e+00 | 1.00e+00 |
| ARFGEF1 | 21 ( 6%) | 5 (3.1%) | 16 (7.7%) | 7.03e-02 | 1.17e-01 |
| PREX2 | 42 (11%) | 13 ( 8.0%) | 29 (13.9%) | 9.79e-02 | 1.55e-01 |
| SULF1 | 25 ( 7%) | 10 (6.2%) | 15 (7.2%) | 8.35e-01 | 8.60e-01 |
| SLCO5A1 | 22 ( 6%) | 5 (3.1%) | 17 (8.2%) | 4.65e-02 | 9.50e-02 |
| PAPPA | 25 ( 7%) | 8 (4.9%) | 17 (8.2%) | 2.97e-01 | 3.60e-01 |
| MPDZ | 23 ( 6%) | 2 ( 1.2%) | 21 (10.1%) | 3.13e-04 | 7.56e-03 |
| SPTAN1 | 20 ( 5%) | 5 (3.1%) | 15 (7.2%) | 1.05e-01 | 1.62e-01 |
| NUP214 | 19 ( 5%) | 3 (1.9%) | 16 (7.7%) | 1.56e-02 | 4.61e-02 |
| NOTCH1 | 21 ( 6%) | 6 (3.7%) | 15 (7.2%) | 1.78e-01 | 2.40e-01 |
| PTPRD | 33 ( 9%) | 12 ( 7.4%) | 21 (10.1%) | 4.63e-01 | 5.15e-01 |
| WWC3 | 19 ( 5%) | 4 (2.5%) | 15 (7.2%) | 5.56e-02 | 1.04e-01 |
| IRS4 | 20 ( 5%) | 4 (2.5%) | 16 (7.7%) | 3.55e-02 | 7.74e-02 |
| TENM1 | 38 (10%) | 11 ( 6.8%) | 27 (13.0%) | 5.83e-02 | 1.05e-01 |
| DCAF12L1 | 20 ( 5%) | 6 (3.7%) | 14 (6.7%) | 2.50e-01 | 3.16e-01 |
| IGSF1 | 19 ( 5%) | 4 (2.5%) | 15 (7.2%) | 5.56e-02 | 1.04e-01 |
| MAGEC1 | 27 ( 7%) | 14 (8.6%) | 13 (6.2%) | 4.24e-01 | 4.76e-01 |
| AFF2 | 27 ( 7%) | 6 ( 3.7%) | 21 (10.1%) | 2.53e-02 | 6.35e-02 |
| BCOR | 30 ( 8%) | 6 ( 3.7%) | 24 (11.5%) | 6.66e-03 | 3.24e-02 |
| HUWE1 | 31 ( 8%) | 5 ( 3.1%) | 26 (12.5%) | 1.07e-03 | 1.45e-02 |
| AR | 19 ( 5%) | 4 (2.5%) | 15 (7.2%) | 5.56e-02 | 1.04e-01 |
| PCDH11X | 27 ( 7%) | 10 (6.2%) | 17 (8.2%) | 5.48e-01 | 5.94e-01 |
| PCDH19 | 27 ( 7%) | 11 (6.8%) | 16 (7.7%) | 8.41e-01 | 8.64e-01 |
| PCDH15 | 61 (16%) | 23 (14.2%) | 38 (18.3%) | 3.25e-01 | 3.92e-01 |
| SFMBT2 | 20 ( 5%) | 5 (3.1%) | 15 (7.2%) | 1.05e-01 | 1.62e-01 |
| NAV2 | 22 ( 6%) | 3 (1.9%) | 19 (9.1%) | 3.27e-03 | 2.00e-02 |
| KCNA4 | 19 ( 5%) | 2 (1.2%) | 17 (8.2%) | 3.24e-03 | 2.00e-02 |
| DLG2 | 21 ( 6%) | 6 (3.7%) | 15 (7.2%) | 1.78e-01 | 2.40e-01 |
| GRM5 | 31 ( 8%) | 7 ( 4.3%) | 24 (11.5%) | 1.38e-02 | 4.47e-02 |
| TMEM132D | 30 ( 8%) | 12 (7.4%) | 18 (8.7%) | 7.05e-01 | 7.47e-01 |
| LRRK2 | 53 (14%) | 14 ( 8.6%) | 39 (18.8%) | 6.80e-03 | 3.28e-02 |
| CD163L1 | 21 ( 6%) | 8 (4.9%) | 13 (6.2%) | 6.56e-01 | 6.99e-01 |
| WNK1 | 25 ( 7%) | 4 ( 2.5%) | 21 (10.1%) | 3.28e-03 | 2.00e-02 |
| PCDH20 | 25 ( 7%) | 5 (3.1%) | 20 (9.6%) | 1.27e-02 | 4.47e-02 |
| MYCBP2 | 37 (10%) | 8 ( 4.9%) | 29 (13.9%) | 4.74e-03 | 2.66e-02 |
| MYO9A | 19 ( 5%) | 7 (4.3%) | 12 (5.8%) | 6.38e-01 | 6.84e-01 |
| NCOR1 | 27 ( 7%) | 5 ( 3.1%) | 22 (10.6%) | 7.77e-03 | 3.49e-02 |
| CPAMD8 | 29 ( 8%) | 12 (7.4%) | 17 (8.2%) | 8.47e-01 | 8.69e-01 |
| COL11A1 | 38 (10%) | 11 ( 6.8%) | 27 (13.0%) | 5.83e-02 | 1.05e-01 |
| TCHH | 40 (11%) | 11 ( 6.8%) | 29 (13.9%) | 2.91e-02 | 7.15e-02 |
| EPRS | 20 ( 5%) | 7 (4.3%) | 13 (6.2%) | 4.92e-01 | 5.36e-01 |
| LYST | 32 ( 9%) | 6 ( 3.7%) | 26 (12.5%) | 2.64e-03 | 2.00e-02 |
| COL24A1 | 21 ( 6%) | 6 (3.7%) | 15 (7.2%) | 1.78e-01 | 2.40e-01 |
| TPTE | 29 ( 8%) | 4 ( 2.5%) | 25 (12.0%) | 6.66e-04 | 1.08e-02 |
| TANC1 | 20 ( 5%) | 4 (2.5%) | 16 (7.7%) | 3.55e-02 | 7.74e-02 |
| CCDC108 | 19 ( 5%) | 3 (1.9%) | 16 (7.7%) | 1.56e-02 | 4.61e-02 |
| DOCK10 | 27 ( 7%) | 6 ( 3.7%) | 21 (10.1%) | 2.53e-02 | 6.35e-02 |
| KALRN | 27 ( 7%) | 3 ( 1.9%) | 24 (11.5%) | 2.21e-04 | 6.72e-03 |
| COL6A6 | 28 ( 8%) | 7 ( 4.3%) | 21 (10.1%) | 4.67e-02 | 9.50e-02 |
| XRN1 | 19 ( 5%) | 2 (1.2%) | 17 (8.2%) | 3.24e-03 | 2.00e-02 |
| MUC4 | 24 ( 6%) | 4 (2.5%) | 20 (9.6%) | 5.35e-03 | 2.83e-02 |
| TRANK1 | 24 ( 6%) | 2 ( 1.2%) | 22 (10.6%) | 1.74e-04 | 6.72e-03 |
| PTPN23 | 20 ( 5%) | 4 (2.5%) | 16 (7.7%) | 3.55e-02 | 7.74e-02 |
| GRM2 | 21 ( 6%) | 5 (3.1%) | 16 (7.7%) | 7.03e-02 | 1.17e-01 |
| ROBO2 | 30 ( 8%) | 7 ( 4.3%) | 23 (11.1%) | 2.09e-02 | 5.71e-02 |
| EPHA5 | 28 ( 8%) | 3 ( 1.9%) | 25 (12.0%) | 2.19e-04 | 6.72e-03 |
| CTNND2 | 32 ( 9%) | 7 ( 4.3%) | 25 (12.0%) | 9.00e-03 | 3.69e-02 |
| PCDHGB3 | 19 ( 5%) | 4 (2.5%) | 15 (7.2%) | 5.56e-02 | 1.04e-01 |
| RXFP3 | 21 ( 6%) | 5 (3.1%) | 16 (7.7%) | 7.03e-02 | 1.17e-01 |
| BDP1 | 21 ( 6%) | 5 (3.1%) | 16 (7.7%) | 7.03e-02 | 1.17e-01 |
| MDC1 | 21 ( 6%) | 4 (2.5%) | 17 (8.2%) | 2.24e-02 | 5.76e-02 |
| TDRD6 | 25 ( 7%) | 3 ( 1.9%) | 22 (10.6%) | 6.56e-04 | 1.08e-02 |
| RIMS1 | 36 (10%) | 10 ( 6.2%) | 26 (12.5%) | 5.13e-02 | 1.04e-01 |
| RELN | 42 (11%) | 9 ( 5.6%) | 33 (15.9%) | 1.68e-03 | 1.79e-02 |
| CNTNAP2 | 33 ( 9%) | 13 (8.0%) | 20 (9.6%) | 7.14e-01 | 7.55e-01 |
| CNTLN | 36 (10%) | 5 ( 3.1%) | 31 (14.9%) | 1.34e-04 | 6.72e-03 |
| TAF1L | 34 ( 9%) | 13 ( 8.0%) | 21 (10.1%) | 5.88e-01 | 6.34e-01 |
| DSCAML1 | 32 ( 9%) | 8 ( 4.9%) | 24 (11.5%) | 2.61e-02 | 6.48e-02 |
| NUAK1 | 19 ( 5%) | 5 (3.1%) | 14 (6.7%) | 1.55e-01 | 2.17e-01 |
| GCN1L1 | 23 ( 6%) | 5 (3.1%) | 18 (8.7%) | 3.05e-02 | 7.31e-02 |
| PRICKLE1 | 19 ( 5%) | 2 (1.2%) | 17 (8.2%) | 3.24e-03 | 2.00e-02 |
| PTPRB | 19 ( 5%) | 3 (1.9%) | 16 (7.7%) | 1.56e-02 | 4.61e-02 |
| NIN | 24 ( 6%) | 5 (3.1%) | 19 (9.1%) | 1.98e-02 | 5.50e-02 |
| SPTB | 22 ( 6%) | 4 (2.5%) | 18 (8.7%) | 1.40e-02 | 4.47e-02 |
| UNC79 | 30 ( 8%) | 10 (6.2%) | 20 (9.6%) | 2.54e-01 | 3.19e-01 |
| ACAN | 26 ( 7%) | 7 (4.3%) | 19 (9.1%) | 9.98e-02 | 1.58e-01 |
| CHD9 | 24 ( 6%) | 8 (4.9%) | 16 (7.7%) | 3.95e-01 | 4.51e-01 |
| MYH2 | 25 ( 7%) | 4 ( 2.5%) | 21 (10.1%) | 3.28e-03 | 2.00e-02 |
| GPR179 | 27 ( 7%) | 6 ( 3.7%) | 21 (10.1%) | 2.53e-02 | 6.35e-02 |
| SDK2 | 19 ( 5%) | 4 (2.5%) | 15 (7.2%) | 5.56e-02 | 1.04e-01 |
| NOTCH3 | 22 ( 6%) | 4 (2.5%) | 18 (8.7%) | 1.40e-02 | 4.47e-02 |
| ZNF536 | 37 (10%) | 11 ( 6.8%) | 26 (12.5%) | 8.12e-02 | 1.32e-01 |
| MEGF8 | 24 ( 6%) | 3 ( 1.9%) | 21 (10.1%) | 1.12e-03 | 1.45e-02 |
| CELSR2 | 25 ( 7%) | 7 (4.3%) | 18 (8.7%) | 1.43e-01 | 2.07e-01 |
| SV2A | 19 ( 5%) | 4 (2.5%) | 15 (7.2%) | 5.56e-02 | 1.04e-01 |
| EPHA2 | 26 ( 7%) | 6 (3.7%) | 20 (9.6%) | 3.86e-02 | 8.21e-02 |
| PIK3C2B | 19 ( 5%) | 3 (1.9%) | 16 (7.7%) | 1.56e-02 | 4.61e-02 |
| MIA3 | 22 ( 6%) | 2 (1.2%) | 20 (9.6%) | 5.64e-04 | 1.08e-02 |
| PCNXL2 | 25 ( 7%) | 8 (4.9%) | 17 (8.2%) | 2.97e-01 | 3.60e-01 |
| FMN2 | 31 ( 8%) | 7 ( 4.3%) | 24 (11.5%) | 1.38e-02 | 4.47e-02 |
| LRRC7 | 28 ( 8%) | 8 (4.9%) | 20 (9.6%) | 1.13e-01 | 1.70e-01 |
| LPHN2 | 21 ( 6%) | 9 (5.6%) | 12 (5.8%) | 1.00e+00 | 1.00e+00 |
| CEP250 | 21 ( 6%) | 1 (0.6%) | 20 (9.6%) | 1.28e-04 | 6.72e-03 |
| DLGAP4 | 20 ( 5%) | 2 (1.2%) | 18 (8.7%) | 1.82e-03 | 1.79e-02 |
| GNAS | 22 ( 6%) | 4 (2.5%) | 18 (8.7%) | 1.40e-02 | 4.47e-02 |
| CDH4 | 19 ( 5%) | 5 (3.1%) | 14 (6.7%) | 1.55e-01 | 2.17e-01 |
| DSCAM | 31 ( 8%) | 6 ( 3.7%) | 25 (12.0%) | 4.20e-03 | 2.51e-02 |
| THSD7B | 30 ( 8%) | 11 (6.8%) | 19 (9.1%) | 4.49e-01 | 5.00e-01 |
| FIGN | 19 ( 5%) | 4 (2.5%) | 15 (7.2%) | 5.56e-02 | 1.04e-01 |
| PLCL1 | 22 ( 6%) | 10 (6.2%) | 12 (5.8%) | 1.00e+00 | 1.00e+00 |
| BMPR2 | 22 ( 6%) | 6 (3.7%) | 16 (7.7%) | 1.24e-01 | 1.85e-01 |
| PARD3B | 20 ( 5%) | 2 (1.2%) | 18 (8.7%) | 1.82e-03 | 1.79e-02 |
| NRXN1 | 39 (10%) | 9 ( 5.6%) | 30 (14.4%) | 6.10e-03 | 3.01e-02 |
| EIF5B | 21 ( 6%) | 4 (2.5%) | 17 (8.2%) | 2.24e-02 | 5.76e-02 |
| STAG1 | 19 ( 5%) | 6 (3.7%) | 13 (6.2%) | 3.45e-01 | 4.07e-01 |
| ATR | 22 ( 6%) | 5 (3.1%) | 17 (8.2%) | 4.65e-02 | 9.50e-02 |
| PCDHA4 | 20 ( 5%) | 6 (3.7%) | 14 (6.7%) | 2.50e-01 | 3.16e-01 |
| PCDHGA12 | 24 ( 6%) | 5 (3.1%) | 19 (9.1%) | 1.98e-02 | 5.50e-02 |
| CDH10 | 29 ( 8%) | 6 ( 3.7%) | 23 (11.1%) | 1.05e-02 | 4.06e-02 |
| MROH2B | 20 ( 5%) | 4 (2.5%) | 16 (7.7%) | 3.55e-02 | 7.74e-02 |
| IGF2R | 21 ( 6%) | 4 (2.5%) | 17 (8.2%) | 2.24e-02 | 5.76e-02 |
| DSP | 26 ( 7%) | 6 (3.7%) | 20 (9.6%) | 3.86e-02 | 8.21e-02 |
| HECW1 | 34 ( 9%) | 5 ( 3.1%) | 29 (13.9%) | 2.29e-04 | 6.72e-03 |
| GRM3 | 22 ( 6%) | 3 (1.9%) | 19 (9.1%) | 3.27e-03 | 2.00e-02 |
| AKAP9 | 33 ( 9%) | 9 ( 5.6%) | 24 (11.5%) | 6.46e-02 | 1.14e-01 |
| SAMD9 | 21 ( 6%) | 7 (4.3%) | 14 (6.7%) | 3.71e-01 | 4.27e-01 |
| LRP12 | 27 ( 7%) | 6 ( 3.7%) | 21 (10.1%) | 2.53e-02 | 6.35e-02 |
| KCNU1 | 21 ( 6%) | 6 (3.7%) | 15 (7.2%) | 1.78e-01 | 2.40e-01 |
| PXDNL | 33 ( 9%) | 10 ( 6.2%) | 23 (11.1%) | 1.40e-01 | 2.06e-01 |
| NPBWR1 | 19 ( 5%) | 4 (2.5%) | 15 (7.2%) | 5.56e-02 | 1.04e-01 |
| NCOA2 | 22 ( 6%) | 7 (4.3%) | 15 (7.2%) | 2.75e-01 | 3.37e-01 |
| GRIN3A | 19 ( 5%) | 3 (1.9%) | 16 (7.7%) | 1.56e-02 | 4.61e-02 |
| ZNF462 | 28 ( 8%) | 5 ( 3.1%) | 23 (11.1%) | 4.78e-03 | 2.66e-02 |
| SETX | 21 ( 6%) | 4 (2.5%) | 17 (8.2%) | 2.24e-02 | 5.76e-02 |
| BCORL1 | 25 ( 7%) | 4 ( 2.5%) | 21 (10.1%) | 3.28e-03 | 2.00e-02 |
| GPR112 | 29 ( 8%) | 6 ( 3.7%) | 23 (11.1%) | 1.05e-02 | 4.06e-02 |
| GPR158 | 22 ( 6%) | 5 (3.1%) | 17 (8.2%) | 4.65e-02 | 9.50e-02 |
| ACACB | 28 ( 8%) | 6 ( 3.7%) | 22 (10.6%) | 1.64e-02 | 4.70e-02 |
| NOS1 | 27 ( 7%) | 5 ( 3.1%) | 22 (10.6%) | 7.77e-03 | 3.49e-02 |
| ITPR2 | 21 ( 6%) | 4 (2.5%) | 17 (8.2%) | 2.24e-02 | 5.76e-02 |
| PZP | 26 ( 7%) | 9 (5.6%) | 17 (8.2%) | 4.14e-01 | 4.66e-01 |
| EDNRB | 29 ( 8%) | 6 ( 3.7%) | 23 (11.1%) | 1.05e-02 | 4.06e-02 |
| ABCC4 | 21 ( 6%) | 5 (3.1%) | 16 (7.7%) | 7.03e-02 | 1.17e-01 |
| KCNH5 | 20 ( 5%) | 5 (3.1%) | 15 (7.2%) | 1.05e-01 | 1.62e-01 |
| NRXN3 | 31 ( 8%) | 4 ( 2.5%) | 27 (13.0%) | 2.22e-04 | 6.72e-03 |
| ADAMTSL3 | 25 ( 7%) | 7 (4.3%) | 18 (8.7%) | 1.43e-01 | 2.07e-01 |
| RNF43 | 32 ( 9%) | 8 ( 4.9%) | 24 (11.5%) | 2.61e-02 | 6.48e-02 |
| SETBP1 | 30 ( 8%) | 10 (6.2%) | 20 (9.6%) | 2.54e-01 | 3.19e-01 |
| ZNF236 | 25 ( 7%) | 6 (3.7%) | 19 (9.1%) | 5.83e-02 | 1.05e-01 |
| ZNF208 | 30 ( 8%) | 11 (6.8%) | 19 (9.1%) | 4.49e-01 | 5.00e-01 |
| PEG3 | 38 (10%) | 6 ( 3.7%) | 32 (15.4%) | 2.10e-04 | 6.72e-03 |
| NTNG1 | 21 ( 6%) | 4 (2.5%) | 17 (8.2%) | 2.24e-02 | 5.76e-02 |
| KCNT2 | 21 ( 6%) | 7 (4.3%) | 14 (6.7%) | 3.71e-01 | 4.27e-01 |
| NFASC | 27 ( 7%) | 4 ( 2.5%) | 23 (11.1%) | 1.96e-03 | 1.79e-02 |
| ACTN2 | 21 ( 6%) | 6 (3.7%) | 15 (7.2%) | 1.78e-01 | 2.40e-01 |
| C1ORF173 | 26 ( 7%) | 10 (6.2%) | 16 (7.7%) | 6.83e-01 | 7.24e-01 |
| TSHZ2 | 20 ( 5%) | 5 (3.1%) | 15 (7.2%) | 1.05e-01 | 1.62e-01 |
| ZDBF2 | 23 ( 6%) | 7 (4.3%) | 16 (7.7%) | 2.01e-01 | 2.65e-01 |
| ABCA12 | 41 (11%) | 10 ( 6.2%) | 31 (14.9%) | 7.73e-03 | 3.49e-02 |
| MYLK | 21 ( 6%) | 5 (3.1%) | 16 (7.7%) | 7.03e-02 | 1.17e-01 |
| NBEAL2 | 23 ( 6%) | 7 (4.3%) | 16 (7.7%) | 2.01e-01 | 2.65e-01 |
| CNTN3 | 22 ( 6%) | 9 (5.6%) | 13 (6.2%) | 8.28e-01 | 8.60e-01 |
| KCND2 | 19 ( 5%) | 6 (3.7%) | 13 (6.2%) | 3.45e-01 | 4.07e-01 |
| PLXNA4 | 41 (11%) | 15 ( 9.3%) | 26 (12.5%) | 4.04e-01 | 4.59e-01 |
| GLI3 | 43 (12%) | 14 ( 8.6%) | 29 (13.9%) | 1.41e-01 | 2.07e-01 |
| NRG1 | 22 ( 6%) | 7 (4.3%) | 15 (7.2%) | 2.75e-01 | 3.37e-01 |
| TRPA1 | 33 ( 9%) | 7 ( 4.3%) | 26 (12.5%) | 5.84e-03 | 2.92e-02 |
| TRPM3 | 22 ( 6%) | 3 (1.9%) | 19 (9.1%) | 3.27e-03 | 2.00e-02 |
| VPS13A | 27 ( 7%) | 7 (4.3%) | 20 (9.6%) | 6.87e-02 | 1.17e-01 |
| NRK | 20 ( 5%) | 3 (1.9%) | 17 (8.2%) | 9.33e-03 | 3.69e-02 |
| NLGN4X | 24 ( 6%) | 6 (3.7%) | 18 (8.7%) | 5.87e-02 | 1.05e-01 |
| DCAF12L2 | 25 ( 7%) | 6 (3.7%) | 19 (9.1%) | 5.83e-02 | 1.05e-01 |
| FLNA | 24 ( 6%) | 4 (2.5%) | 20 (9.6%) | 5.35e-03 | 2.83e-02 |
| CTNND1 | 20 ( 5%) | 6 (3.7%) | 14 (6.7%) | 2.50e-01 | 3.16e-01 |
| BPTF | 22 ( 6%) | 2 (1.2%) | 20 (9.6%) | 5.64e-04 | 1.08e-02 |
| ASXL3 | 20 ( 5%) | 6 (3.7%) | 14 (6.7%) | 2.50e-01 | 3.16e-01 |
| DSEL | 27 ( 7%) | 8 (4.9%) | 19 (9.1%) | 1.59e-01 | 2.22e-01 |
| CACNA1A | 22 ( 6%) | 3 (1.9%) | 19 (9.1%) | 3.27e-03 | 2.00e-02 |
| LPHN3 | 22 ( 6%) | 7 (4.3%) | 15 (7.2%) | 2.75e-01 | 3.37e-01 |
| FBN2 | 32 ( 9%) | 9 ( 5.6%) | 23 (11.1%) | 6.51e-02 | 1.14e-01 |
| ZNF804B | 29 ( 8%) | 7 ( 4.3%) | 22 (10.6%) | 3.14e-02 | 7.43e-02 |
| DLGAP2 | 21 ( 6%) | 7 (4.3%) | 14 (6.7%) | 3.71e-01 | 4.27e-01 |
| COL27A1 | 19 ( 5%) | 3 (1.9%) | 16 (7.7%) | 1.56e-02 | 4.61e-02 |
| FRY | 29 ( 8%) | 7 ( 4.3%) | 22 (10.6%) | 3.14e-02 | 7.43e-02 |
| ZNF43 | 23 ( 6%) | 6 (3.7%) | 17 (8.2%) | 8.60e-02 | 1.37e-01 |
| SPTA1 | 67 (18%) | 25 (15.4%) | 42 (20.2%) | 2.77e-01 | 3.39e-01 |
| SCN9A | 32 ( 9%) | 11 ( 6.8%) | 21 (10.1%) | 3.51e-01 | 4.13e-01 |
| CUL9 | 25 ( 7%) | 6 (3.7%) | 19 (9.1%) | 5.83e-02 | 1.05e-01 |
| MXRA5 | 28 ( 8%) | 5 ( 3.1%) | 23 (11.1%) | 4.78e-03 | 2.66e-02 |
| CPXM2 | 23 ( 6%) | 7 (4.3%) | 16 (7.7%) | 2.01e-01 | 2.65e-01 |
| KIAA1217 | 22 ( 6%) | 3 (1.9%) | 19 (9.1%) | 3.27e-03 | 2.00e-02 |
| PTCHD3 | 19 ( 5%) | 3 (1.9%) | 16 (7.7%) | 1.56e-02 | 4.61e-02 |
| JMJD1C | 20 ( 5%) | 5 (3.1%) | 15 (7.2%) | 1.05e-01 | 1.62e-01 |
| TET1 | 19 ( 5%) | 3 (1.9%) | 16 (7.7%) | 1.56e-02 | 4.61e-02 |
| ITIH5 | 24 ( 6%) | 7 (4.3%) | 17 (8.2%) | 2.01e-01 | 2.65e-01 |
| KCNMA1 | 24 ( 6%) | 2 ( 1.2%) | 22 (10.6%) | 1.74e-04 | 6.72e-03 |
| NRG3 | 23 ( 6%) | 8 (4.9%) | 15 (7.2%) | 3.96e-01 | 4.51e-01 |
| LARP4B | 26 ( 7%) | 5 ( 3.1%) | 21 (10.1%) | 1.25e-02 | 4.47e-02 |
| NPAT | 19 ( 5%) | 6 (3.7%) | 13 (6.2%) | 3.45e-01 | 4.07e-01 |
| IGSF9B | 20 ( 5%) | 3 (1.9%) | 17 (8.2%) | 9.33e-03 | 3.69e-02 |
| NUP98 | 19 ( 5%) | 4 (2.5%) | 15 (7.2%) | 5.56e-02 | 1.04e-01 |
| NRXN2 | 24 ( 6%) | 3 ( 1.9%) | 21 (10.1%) | 1.12e-03 | 1.45e-02 |
| SPTBN2 | 20 ( 5%) | 7 (4.3%) | 13 (6.2%) | 4.92e-01 | 5.36e-01 |
| NUMA1 | 24 ( 6%) | 3 ( 1.9%) | 21 (10.1%) | 1.12e-03 | 1.45e-02 |
| KSR2 | 20 ( 5%) | 3 (1.9%) | 17 (8.2%) | 9.33e-03 | 3.69e-02 |
| GOLGA3 | 21 ( 6%) | 2 (1.2%) | 19 (9.1%) | 1.01e-03 | 1.44e-02 |
| CACNA1C | 31 ( 8%) | 7 ( 4.3%) | 24 (11.5%) | 1.38e-02 | 4.47e-02 |
| KIF21A | 22 ( 6%) | 5 (3.1%) | 17 (8.2%) | 4.65e-02 | 9.50e-02 |
| CNTN1 | 19 ( 5%) | 4 (2.5%) | 15 (7.2%) | 5.56e-02 | 1.04e-01 |
| PDZRN4 | 20 ( 5%) | 5 (3.1%) | 15 (7.2%) | 1.05e-01 | 1.62e-01 |
| KCNA5 | 19 ( 5%) | 7 (4.3%) | 12 (5.8%) | 6.38e-01 | 6.84e-01 |
| ERBB3 | 35 (10%) | 13 ( 8.0%) | 22 (10.6%) | 4.76e-01 | 5.29e-01 |
| CEP290 | 19 ( 5%) | 2 (1.2%) | 17 (8.2%) | 3.24e-03 | 2.00e-02 |
| C12ORF55 | 19 ( 5%) | 8 (4.9%) | 11 (5.3%) | 1.00e+00 | 1.00e+00 |
| COL4A1 | 22 ( 6%) | 4 (2.5%) | 18 (8.7%) | 1.40e-02 | 4.47e-02 |
| SPG20 | 22 ( 6%) | 7 (4.3%) | 15 (7.2%) | 2.75e-01 | 3.37e-01 |
| SLITRK1 | 29 ( 8%) | 8 ( 4.9%) | 21 (10.1%) | 7.99e-02 | 1.30e-01 |
| SLITRK6 | 22 ( 6%) | 7 (4.3%) | 15 (7.2%) | 2.75e-01 | 3.37e-01 |
| DYNC1H1 | 35 (10%) | 7 ( 4.3%) | 28 (13.5%) | 3.64e-03 | 2.20e-02 |
| HECTD1 | 20 ( 5%) | 6 (3.7%) | 14 (6.7%) | 2.50e-01 | 3.16e-01 |
| FANCM | 20 ( 5%) | 7 (4.3%) | 13 (6.2%) | 4.92e-01 | 5.36e-01 |
| MKRN3 | 19 ( 5%) | 6 (3.7%) | 13 (6.2%) | 3.45e-01 | 4.07e-01 |
| TRPM1 | 19 ( 5%) | 3 (1.9%) | 16 (7.7%) | 1.56e-02 | 4.61e-02 |
| DUOX2 | 19 ( 5%) | 4 (2.5%) | 15 (7.2%) | 5.56e-02 | 1.04e-01 |
| SLC12A1 | 21 ( 6%) | 7 (4.3%) | 14 (6.7%) | 3.71e-01 | 4.27e-01 |
| FBN1 | 34 ( 9%) | 7 ( 4.3%) | 27 (13.0%) | 5.79e-03 | 2.92e-02 |
| CSPG4 | 23 ( 6%) | 6 (3.7%) | 17 (8.2%) | 8.60e-02 | 1.37e-01 |
| AGBL1 | 20 ( 5%) | 4 (2.5%) | 16 (7.7%) | 3.55e-02 | 7.74e-02 |
| RBBP6 | 19 ( 5%) | 6 (3.7%) | 13 (6.2%) | 3.45e-01 | 4.07e-01 |
| TNRC6A | 23 ( 6%) | 8 (4.9%) | 15 (7.2%) | 3.96e-01 | 4.51e-01 |
| CNOT1 | 25 ( 7%) | 5 (3.1%) | 20 (9.6%) | 1.27e-02 | 4.47e-02 |
| WDR90 | 19 ( 5%) | 6 (3.7%) | 13 (6.2%) | 3.45e-01 | 4.07e-01 |
| CNTNAP4 | 20 ( 5%) | 5 (3.1%) | 15 (7.2%) | 1.05e-01 | 1.62e-01 |
| MYH3 | 21 ( 6%) | 5 (3.1%) | 16 (7.7%) | 7.03e-02 | 1.17e-01 |
| KIAA0100 | 24 ( 6%) | 5 (3.1%) | 19 (9.1%) | 1.98e-02 | 5.50e-02 |
| ACACA | 26 ( 7%) | 8 (4.9%) | 18 (8.7%) | 2.19e-01 | 2.85e-01 |
| CNTNAP1 | 21 ( 6%) | 2 (1.2%) | 19 (9.1%) | 1.01e-03 | 1.44e-02 |
| ABCA10 | 20 ( 5%) | 4 (2.5%) | 16 (7.7%) | 3.55e-02 | 7.74e-02 |
| MYH10 | 30 ( 8%) | 7 ( 4.3%) | 23 (11.1%) | 2.09e-02 | 5.71e-02 |
| FHOD3 | 25 ( 7%) | 2 ( 1.2%) | 23 (11.1%) | 9.62e-05 | 6.72e-03 |
| EPG5 | 25 ( 7%) | 4 ( 2.5%) | 21 (10.1%) | 3.28e-03 | 2.00e-02 |
| SMAD4 | 30 ( 8%) | 13 (8.0%) | 17 (8.2%) | 1.00e+00 | 1.00e+00 |
| DCC | 29 ( 8%) | 8 ( 4.9%) | 21 (10.1%) | 7.99e-02 | 1.30e-01 |
| EPB41L3 | 28 ( 8%) | 8 (4.9%) | 20 (9.6%) | 1.13e-01 | 1.70e-01 |
| ZNF516 | 19 ( 5%) | 1 (0.6%) | 18 (8.7%) | 2.40e-04 | 6.72e-03 |
| ANKRD12 | 19 ( 5%) | 3 (1.9%) | 16 (7.7%) | 1.56e-02 | 4.61e-02 |
| NCAN | 21 ( 6%) | 7 (4.3%) | 14 (6.7%) | 3.71e-01 | 4.27e-01 |
| ATP4A | 20 ( 5%) | 4 (2.5%) | 16 (7.7%) | 3.55e-02 | 7.74e-02 |
| CIC | 24 ( 6%) | 6 (3.7%) | 18 (8.7%) | 5.87e-02 | 1.05e-01 |
| PTPRS | 24 ( 6%) | 5 (3.1%) | 19 (9.1%) | 1.98e-02 | 5.50e-02 |
| MAP2K7 | 21 ( 6%) | 8 (4.9%) | 13 (6.2%) | 6.56e-01 | 6.99e-01 |
| CASZ1 | 19 ( 5%) | 4 (2.5%) | 15 (7.2%) | 5.56e-02 | 1.04e-01 |
| MTOR | 24 ( 6%) | 7 (4.3%) | 17 (8.2%) | 2.01e-01 | 2.65e-01 |
| PTCHD2 | 21 ( 6%) | 5 (3.1%) | 16 (7.7%) | 7.03e-02 | 1.17e-01 |
| NOTCH2 | 20 ( 5%) | 2 (1.2%) | 18 (8.7%) | 1.82e-03 | 1.79e-02 |
| PRDM2 | 23 ( 6%) | 8 (4.9%) | 15 (7.2%) | 3.96e-01 | 4.51e-01 |
| POGZ | 21 ( 6%) | 6 (3.7%) | 15 (7.2%) | 1.78e-01 | 2.40e-01 |
| FLG2 | 29 ( 8%) | 9 (5.6%) | 20 (9.6%) | 1.75e-01 | 2.40e-01 |
| SPEN | 33 ( 9%) | 9 ( 5.6%) | 24 (11.5%) | 6.46e-02 | 1.14e-01 |
| TNN | 26 ( 7%) | 4 ( 2.5%) | 22 (10.6%) | 2.00e-03 | 1.79e-02 |
| ASTN1 | 24 ( 6%) | 11 (6.8%) | 13 (6.2%) | 8.35e-01 | 8.60e-01 |
| CAMSAP2 | 20 ( 5%) | 2 (1.2%) | 18 (8.7%) | 1.82e-03 | 1.79e-02 |
| CACNA1S | 21 ( 6%) | 6 (3.7%) | 15 (7.2%) | 1.78e-01 | 2.40e-01 |
| NID1 | 22 ( 6%) | 7 (4.3%) | 15 (7.2%) | 2.75e-01 | 3.37e-01 |
| AHCTF1 | 19 ( 5%) | 3 (1.9%) | 16 (7.7%) | 1.56e-02 | 4.61e-02 |
| CSMD2 | 36 (10%) | 7 ( 4.3%) | 29 (13.9%) | 2.27e-03 | 1.99e-02 |
| SZT2 | 21 ( 6%) | 6 (3.7%) | 15 (7.2%) | 1.78e-01 | 2.40e-01 |
| RPL22 | 28 ( 8%) | 6 ( 3.7%) | 22 (10.6%) | 1.64e-02 | 4.70e-02 |
| CAMTA1 | 19 ( 5%) | 5 (3.1%) | 14 (6.7%) | 1.55e-01 | 2.17e-01 |
| HFM1 | 19 ( 5%) | 5 (3.1%) | 14 (6.7%) | 1.55e-01 | 2.17e-01 |
| MYH7B | 23 ( 6%) | 2 ( 1.2%) | 21 (10.1%) | 3.13e-04 | 7.56e-03 |
| CHD6 | 32 ( 9%) | 6 ( 3.7%) | 26 (12.5%) | 2.64e-03 | 2.00e-02 |
| PTPRT | 45 (12%) | 14 ( 8.6%) | 31 (14.9%) | 7.83e-02 | 1.29e-01 |
| ARFGEF2 | 24 ( 6%) | 4 (2.5%) | 20 (9.6%) | 5.35e-03 | 2.83e-02 |
| ZNF831 | 23 ( 6%) | 5 (3.1%) | 18 (8.7%) | 3.05e-02 | 7.31e-02 |
| DOPEY2 | 22 ( 6%) | 4 (2.5%) | 18 (8.7%) | 1.40e-02 | 4.47e-02 |
| MYO18B | 21 ( 6%) | 2 (1.2%) | 19 (9.1%) | 1.01e-03 | 1.44e-02 |
| MYH9 | 25 ( 7%) | 8 (4.9%) | 17 (8.2%) | 2.97e-01 | 3.60e-01 |
| NCKAP5 | 28 ( 8%) | 8 (4.9%) | 20 (9.6%) | 1.13e-01 | 1.70e-01 |
| NBAS | 20 ( 5%) | 6 (3.7%) | 14 (6.7%) | 2.50e-01 | 3.16e-01 |
| SCN3A | 28 ( 8%) | 5 ( 3.1%) | 23 (11.1%) | 4.78e-03 | 2.66e-02 |
| SCN2A | 27 ( 7%) | 6 ( 3.7%) | 21 (10.1%) | 2.53e-02 | 6.35e-02 |
| SCN1A | 26 ( 7%) | 6 (3.7%) | 20 (9.6%) | 3.86e-02 | 8.21e-02 |
| IRS1 | 19 ( 5%) | 5 (3.1%) | 14 (6.7%) | 1.55e-01 | 2.17e-01 |
| COL4A4 | 19 ( 5%) | 5 (3.1%) | 14 (6.7%) | 1.55e-01 | 2.17e-01 |
| AGAP1 | 24 ( 6%) | 6 (3.7%) | 18 (8.7%) | 5.87e-02 | 1.05e-01 |
| XDH | 23 ( 6%) | 6 (3.7%) | 17 (8.2%) | 8.60e-02 | 1.37e-01 |
| BIRC6 | 30 ( 8%) | 7 ( 4.3%) | 23 (11.1%) | 2.09e-02 | 5.71e-02 |
| TET3 | 19 ( 5%) | 2 (1.2%) | 17 (8.2%) | 3.24e-03 | 2.00e-02 |
| KIDINS220 | 21 ( 6%) | 4 (2.5%) | 17 (8.2%) | 2.24e-02 | 5.76e-02 |
| ZBTB20 | 37 (10%) | 10 ( 6.2%) | 27 (13.0%) | 3.58e-02 | 7.78e-02 |
| PLXNA1 | 24 ( 6%) | 3 ( 1.9%) | 21 (10.1%) | 1.12e-03 | 1.45e-02 |
| SI | 31 ( 8%) | 9 ( 5.6%) | 22 (10.6%) | 9.16e-02 | 1.46e-01 |
| KCNH8 | 22 ( 6%) | 8 (4.9%) | 14 (6.7%) | 5.14e-01 | 5.58e-01 |
| ITPR1 | 24 ( 6%) | 8 (4.9%) | 16 (7.7%) | 3.95e-01 | 4.51e-01 |
| DOCK3 | 41 (11%) | 10 ( 6.2%) | 31 (14.9%) | 7.73e-03 | 3.49e-02 |
| CACNA2D3 | 23 ( 6%) | 4 (2.5%) | 19 (9.1%) | 8.69e-03 | 3.69e-02 |
| FLNB | 23 ( 6%) | 4 (2.5%) | 19 (9.1%) | 8.69e-03 | 3.69e-02 |
| GRM7 | 23 ( 6%) | 11 (6.8%) | 12 (5.8%) | 8.29e-01 | 8.60e-01 |
| EPHA3 | 25 ( 7%) | 4 ( 2.5%) | 21 (10.1%) | 3.28e-03 | 2.00e-02 |
| BOD1L1 | 24 ( 6%) | 6 (3.7%) | 18 (8.7%) | 5.87e-02 | 1.05e-01 |
| PCDH18 | 24 ( 6%) | 5 (3.1%) | 19 (9.1%) | 1.98e-02 | 5.50e-02 |
| LRBA | 21 ( 6%) | 5 (3.1%) | 16 (7.7%) | 7.03e-02 | 1.17e-01 |
| GRIA2 | 19 ( 5%) | 8 (4.9%) | 11 (5.3%) | 1.00e+00 | 1.00e+00 |
| FSTL5 | 22 ( 6%) | 4 (2.5%) | 18 (8.7%) | 1.40e-02 | 4.47e-02 |
| SLIT2 | 30 ( 8%) | 9 ( 5.6%) | 21 (10.1%) | 1.27e-01 | 1.88e-01 |
| FRYL | 20 ( 5%) | 7 (4.3%) | 13 (6.2%) | 4.92e-01 | 5.36e-01 |
| EVC | 25 ( 7%) | 6 (3.7%) | 19 (9.1%) | 5.83e-02 | 1.05e-01 |
| WDFY3 | 35 (10%) | 8 ( 4.9%) | 27 (13.0%) | 1.13e-02 | 4.32e-02 |
| PTPN13 | 23 ( 6%) | 2 ( 1.2%) | 21 (10.1%) | 3.13e-04 | 7.56e-03 |
| APC | 39 (10%) | 8 ( 4.9%) | 31 (14.9%) | 1.92e-03 | 1.79e-02 |
| PCDHA8 | 28 ( 8%) | 8 (4.9%) | 20 (9.6%) | 1.13e-01 | 1.70e-01 |
| PCDHA12 | 26 ( 7%) | 9 (5.6%) | 17 (8.2%) | 4.14e-01 | 4.66e-01 |
| PCDHA13 | 22 ( 6%) | 7 (4.3%) | 15 (7.2%) | 2.75e-01 | 3.37e-01 |
| TRIO | 31 ( 8%) | 7 ( 4.3%) | 24 (11.5%) | 1.38e-02 | 4.47e-02 |
| DOCK2 | 40 (11%) | 8 ( 4.9%) | 32 (15.4%) | 1.21e-03 | 1.54e-02 |
| CDH12 | 24 ( 6%) | 7 (4.3%) | 17 (8.2%) | 2.01e-01 | 2.65e-01 |
| CDH9 | 27 ( 7%) | 6 ( 3.7%) | 21 (10.1%) | 2.53e-02 | 6.35e-02 |
| NIPBL | 41 (11%) | 11 ( 6.8%) | 30 (14.4%) | 2.90e-02 | 7.15e-02 |
| C5ORF42 | 29 ( 8%) | 8 ( 4.9%) | 21 (10.1%) | 7.99e-02 | 1.30e-01 |
| KIAA0947 | 24 ( 6%) | 5 (3.1%) | 19 (9.1%) | 1.98e-02 | 5.50e-02 |
| MAP1B | 22 ( 6%) | 8 (4.9%) | 14 (6.7%) | 5.14e-01 | 5.58e-01 |
| SOGA3 | 19 ( 5%) | 8 (4.9%) | 11 (5.3%) | 1.00e+00 | 1.00e+00 |
| BCLAF1 | 25 ( 7%) | 7 (4.3%) | 18 (8.7%) | 1.43e-01 | 2.07e-01 |
| HIVEP2 | 23 ( 6%) | 3 (1.9%) | 20 (9.6%) | 1.92e-03 | 1.79e-02 |
| JARID2 | 25 ( 7%) | 5 (3.1%) | 20 (9.6%) | 1.27e-02 | 4.47e-02 |
| ARID1B | 25 ( 7%) | 5 (3.1%) | 20 (9.6%) | 1.27e-02 | 4.47e-02 |
| TULP4 | 19 ( 5%) | 8 (4.9%) | 11 (5.3%) | 1.00e+00 | 1.00e+00 |
| DHX16 | 20 ( 5%) | 3 (1.9%) | 17 (8.2%) | 9.33e-03 | 3.69e-02 |
| NOTCH4 | 23 ( 6%) | 6 (3.7%) | 17 (8.2%) | 8.60e-02 | 1.37e-01 |
| ITPR3 | 29 ( 8%) | 7 ( 4.3%) | 22 (10.6%) | 3.14e-02 | 7.43e-02 |
| TRERF1 | 19 ( 5%) | 5 (3.1%) | 14 (6.7%) | 1.55e-01 | 2.17e-01 |
| PHF3 | 20 ( 5%) | 7 (4.3%) | 13 (6.2%) | 4.92e-01 | 5.36e-01 |
| ZNF292 | 24 ( 6%) | 4 (2.5%) | 20 (9.6%) | 5.35e-03 | 2.83e-02 |
| KMT2E | 25 ( 7%) | 7 (4.3%) | 18 (8.7%) | 1.43e-01 | 2.07e-01 |
| DOCK4 | 24 ( 6%) | 8 (4.9%) | 16 (7.7%) | 3.95e-01 | 4.51e-01 |
| PPP1R3A | 20 ( 5%) | 6 (3.7%) | 14 (6.7%) | 2.50e-01 | 3.16e-01 |
| NUP205 | 23 ( 6%) | 4 (2.5%) | 19 (9.1%) | 8.69e-03 | 3.69e-02 |
| KIAA1549 | 20 ( 5%) | 6 (3.7%) | 14 (6.7%) | 2.50e-01 | 3.16e-01 |
| CARD11 | 25 ( 7%) | 4 ( 2.5%) | 21 (10.1%) | 3.28e-03 | 2.00e-02 |
| SDK1 | 48 (13%) | 15 ( 9.3%) | 33 (15.9%) | 6.33e-02 | 1.13e-01 |
| ABCB4 | 23 ( 6%) | 5 (3.1%) | 18 (8.7%) | 3.05e-02 | 7.31e-02 |
| COL1A2 | 26 ( 7%) | 9 (5.6%) | 17 (8.2%) | 4.14e-01 | 4.66e-01 |
| COL14A1 | 26 ( 7%) | 4 ( 2.5%) | 22 (10.6%) | 2.00e-03 | 1.79e-02 |
| ZFAT | 20 ( 5%) | 4 (2.5%) | 16 (7.7%) | 3.55e-02 | 7.74e-02 |
| DENND3 | 20 ( 5%) | 4 (2.5%) | 16 (7.7%) | 3.55e-02 | 7.74e-02 |
| UNC5D | 20 ( 5%) | 7 (4.3%) | 13 (6.2%) | 4.92e-01 | 5.36e-01 |
| KAT6A | 25 ( 7%) | 4 ( 2.5%) | 21 (10.1%) | 3.28e-03 | 2.00e-02 |
| RP1 | 40 (11%) | 13 ( 8%) | 27 (13%) | 1.33e-01 | 1.97e-01 |
| SVEP1 | 29 ( 8%) | 5 ( 3.1%) | 24 (11.5%) | 2.92e-03 | 2.00e-02 |
| TNC | 20 ( 5%) | 7 (4.3%) | 13 (6.2%) | 4.92e-01 | 5.36e-01 |
| ASTN2 | 28 ( 8%) | 6 ( 3.7%) | 22 (10.6%) | 1.64e-02 | 4.70e-02 |
| TLR4 | 26 ( 7%) | 9 (5.6%) | 17 (8.2%) | 4.14e-01 | 4.66e-01 |
| CDK5RAP2 | 20 ( 5%) | 9 (5.6%) | 11 (5.3%) | 1.00e+00 | 1.00e+00 |
| PRRC2B | 21 ( 6%) | 7 (4.3%) | 14 (6.7%) | 3.71e-01 | 4.27e-01 |
| COL5A1 | 26 ( 7%) | 5 ( 3.1%) | 21 (10.1%) | 1.25e-02 | 4.47e-02 |
| ABCA2 | 24 ( 6%) | 7 (4.3%) | 17 (8.2%) | 2.01e-01 | 2.65e-01 |
| CACNA1B | 27 ( 7%) | 4 ( 2.5%) | 23 (11.1%) | 1.96e-03 | 1.79e-02 |
| ADAMTSL1 | 19 ( 5%) | 6 (3.7%) | 13 (6.2%) | 3.45e-01 | 4.07e-01 |
| SMARCA2 | 23 ( 6%) | 4 (2.5%) | 19 (9.1%) | 8.69e-03 | 3.69e-02 |
| PLXNA3 | 19 ( 5%) | 4 (2.5%) | 15 (7.2%) | 5.56e-02 | 1.04e-01 |
| F8 | 26 ( 7%) | 5 ( 3.1%) | 21 (10.1%) | 1.25e-02 | 4.47e-02 |
| DMD | 51 (14%) | 12 ( 7.4%) | 39 (18.8%) | 2.12e-03 | 1.88e-02 |
| FAM47C | 22 ( 6%) | 4 (2.5%) | 18 (8.7%) | 1.40e-02 | 4.47e-02 |
| PLXNC1 | 19 ( 5%) | 4 (2.5%) | 15 (7.2%) | 5.56e-02 | 1.04e-01 |
| NBEA | 48 (13%) | 11 ( 6.8%) | 37 (17.8%) | 1.72e-03 | 1.79e-02 |
| CR1 | 20 ( 5%) | 6 (3.7%) | 14 (6.7%) | 2.50e-01 | 3.16e-01 |
| CTNNA2 | 22 ( 6%) | 6 (3.7%) | 16 (7.7%) | 1.24e-01 | 1.85e-01 |
| EVC2 | 26 ( 7%) | 9 (5.6%) | 17 (8.2%) | 4.14e-01 | 4.66e-01 |
| DMXL1 | 22 ( 6%) | 4 (2.5%) | 18 (8.7%) | 1.40e-02 | 4.47e-02 |
| DMXL2 | 23 ( 6%) | 7 (4.3%) | 16 (7.7%) | 2.01e-01 | 2.65e-01 |
| ERBB2 | 21 ( 6%) | 9 (5.6%) | 12 (5.8%) | 1.00e+00 | 1.00e+00 |
| BCL9 | 20 ( 5%) | 2 (1.2%) | 18 (8.7%) | 1.82e-03 | 1.79e-02 |
| ADAMTS16 | 33 ( 9%) | 9 ( 5.6%) | 24 (11.5%) | 6.46e-02 | 1.14e-01 |
| SORCS1 | 26 ( 7%) | 8 (4.9%) | 18 (8.7%) | 2.19e-01 | 2.85e-01 |
| MYO3A | 25 ( 7%) | 12 (7.4%) | 13 (6.2%) | 6.81e-01 | 7.23e-01 |
| CNTN5 | 22 ( 6%) | 7 (4.3%) | 15 (7.2%) | 2.75e-01 | 3.37e-01 |
| KIAA1549L | 19 ( 5%) | 3 (1.9%) | 16 (7.7%) | 1.56e-02 | 4.61e-02 |
| OR5M3 | 20 ( 5%) | 4 (2.5%) | 16 (7.7%) | 3.55e-02 | 7.74e-02 |
| C2CD3 | 20 ( 5%) | 4 (2.5%) | 16 (7.7%) | 3.55e-02 | 7.74e-02 |
| MBD6 | 19 ( 5%) | 4 (2.5%) | 15 (7.2%) | 5.56e-02 | 1.04e-01 |
| ZC3H13 | 28 ( 8%) | 7 ( 4.3%) | 21 (10.1%) | 4.67e-02 | 9.50e-02 |
| ZNF106 | 21 ( 6%) | 7 (4.3%) | 14 (6.7%) | 3.71e-01 | 4.27e-01 |
| CACNA1H | 23 ( 6%) | 5 (3.1%) | 18 (8.7%) | 3.05e-02 | 7.31e-02 |
| ADAMTS18 | 29 ( 8%) | 9 (5.6%) | 20 (9.6%) | 1.75e-01 | 2.40e-01 |
| RAI1 | 23 ( 6%) | 3 (1.9%) | 20 (9.6%) | 1.92e-03 | 1.79e-02 |
| SSH2 | 20 ( 5%) | 6 (3.7%) | 14 (6.7%) | 2.50e-01 | 3.16e-01 |
| ZNF407 | 22 ( 6%) | 3 (1.9%) | 19 (9.1%) | 3.27e-03 | 2.00e-02 |
| PRG4 | 20 ( 5%) | 2 (1.2%) | 18 (8.7%) | 1.82e-03 | 1.79e-02 |
| TPR | 19 ( 5%) | 4 (2.5%) | 15 (7.2%) | 5.56e-02 | 1.04e-01 |
| BRINP3 | 21 ( 6%) | 8 (4.9%) | 13 (6.2%) | 6.56e-01 | 6.99e-01 |
| SIPA1L2 | 25 ( 7%) | 6 (3.7%) | 19 (9.1%) | 5.83e-02 | 1.05e-01 |
| DPYD | 20 ( 5%) | 7 (4.3%) | 13 (6.2%) | 4.92e-01 | 5.36e-01 |
| PAX1 | 20 ( 5%) | 3 (1.9%) | 17 (8.2%) | 9.33e-03 | 3.69e-02 |
| GREB1 | 24 ( 6%) | 6 (3.7%) | 18 (8.7%) | 5.87e-02 | 1.05e-01 |
| GLI2 | 21 ( 6%) | 2 (1.2%) | 19 (9.1%) | 1.01e-03 | 1.44e-02 |
| MAP2 | 40 (11%) | 11 ( 6.8%) | 29 (13.9%) | 2.91e-02 | 7.15e-02 |
| CPS1 | 20 ( 5%) | 7 (4.3%) | 13 (6.2%) | 4.92e-01 | 5.36e-01 |
| FSHR | 21 ( 6%) | 6 (3.7%) | 15 (7.2%) | 1.78e-01 | 2.40e-01 |
| SCN11A | 29 ( 8%) | 5 ( 3.1%) | 24 (11.5%) | 2.92e-03 | 2.00e-02 |
| RBM6 | 21 ( 6%) | 3 (1.9%) | 18 (8.7%) | 5.54e-03 | 2.83e-02 |
| PCDHA7 | 25 ( 7%) | 7 (4.3%) | 18 (8.7%) | 1.43e-01 | 2.07e-01 |
| PCDHGA6 | 19 ( 5%) | 5 (3.1%) | 14 (6.7%) | 1.55e-01 | 2.17e-01 |
| ADAMTS12 | 28 ( 8%) | 8 (4.9%) | 20 (9.6%) | 1.13e-01 | 1.70e-01 |
| RICTOR | 20 ( 5%) | 4 (2.5%) | 16 (7.7%) | 3.55e-02 | 7.74e-02 |
| TTK | 20 ( 5%) | 8 (4.9%) | 12 (5.8%) | 8.19e-01 | 8.57e-01 |
| WBSCR17 | 25 ( 7%) | 10 (6.2%) | 15 (7.2%) | 8.35e-01 | 8.60e-01 |
| FER1L6 | 23 ( 6%) | 4 (2.5%) | 19 (9.1%) | 8.69e-03 | 3.69e-02 |
| XPO7 | 19 ( 5%) | 4 (2.5%) | 15 (7.2%) | 5.56e-02 | 1.04e-01 |
| CHD7 | 33 ( 9%) | 10 ( 6.2%) | 23 (11.1%) | 1.40e-01 | 2.06e-01 |
| MMP16 | 20 ( 5%) | 4 (2.5%) | 16 (7.7%) | 3.55e-02 | 7.74e-02 |
| PGM5 | 25 ( 7%) | 7 (4.3%) | 18 (8.7%) | 1.43e-01 | 2.07e-01 |
| ZEB2 | 21 ( 6%) | 7 (4.3%) | 14 (6.7%) | 3.71e-01 | 4.27e-01 |
| SPEF2 | 28 ( 8%) | 6 ( 3.7%) | 22 (10.6%) | 1.64e-02 | 4.70e-02 |
| SBF1 | 23 ( 6%) | 5 (3.1%) | 18 (8.7%) | 3.05e-02 | 7.31e-02 |
| KIAA1462 | 21 ( 6%) | 5 (3.1%) | 16 (7.7%) | 7.03e-02 | 1.17e-01 |
| MTUS2 | 21 ( 6%) | 4 (2.5%) | 17 (8.2%) | 2.24e-02 | 5.76e-02 |
| ARHGAP5 | 23 ( 6%) | 6 (3.7%) | 17 (8.2%) | 8.60e-02 | 1.37e-01 |
| SALL3 | 20 ( 5%) | 8 (4.9%) | 12 (5.8%) | 8.19e-01 | 8.57e-01 |
| WDR47 | 19 ( 5%) | 5 (3.1%) | 14 (6.7%) | 1.55e-01 | 2.17e-01 |
| ADCY10 | 22 ( 6%) | 7 (4.3%) | 15 (7.2%) | 2.75e-01 | 3.37e-01 |
| SEMA5A | 22 ( 6%) | 3 (1.9%) | 19 (9.1%) | 3.27e-03 | 2.00e-02 |
| LRRN3 | 20 ( 5%) | 6 (3.7%) | 14 (6.7%) | 2.50e-01 | 3.16e-01 |
| ZFPM2 | 19 ( 5%) | 4 (2.5%) | 15 (7.2%) | 5.56e-02 | 1.04e-01 |
| TLN1 | 19 ( 5%) | 6 (3.7%) | 13 (6.2%) | 3.45e-01 | 4.07e-01 |
| SLITRK2 | 26 ( 7%) | 9 (5.6%) | 17 (8.2%) | 4.14e-01 | 4.66e-01 |
| USP9X | 22 ( 6%) | 8 (4.9%) | 14 (6.7%) | 5.14e-01 | 5.58e-01 |
| ATM | 38 (10%) | 11 ( 6.8%) | 27 (13.0%) | 5.83e-02 | 1.05e-01 |
| OCA2 | 21 ( 6%) | 5 (3.1%) | 16 (7.7%) | 7.03e-02 | 1.17e-01 |
| ZNF423 | 19 ( 5%) | 6 (3.7%) | 13 (6.2%) | 3.45e-01 | 4.07e-01 |
| ASH1L | 33 ( 9%) | 9 ( 5.6%) | 24 (11.5%) | 6.46e-02 | 1.14e-01 |
| NCAM2 | 23 ( 6%) | 6 (3.7%) | 17 (8.2%) | 8.60e-02 | 1.37e-01 |
| ATP2B2 | 19 ( 5%) | 2 (1.2%) | 17 (8.2%) | 3.24e-03 | 2.00e-02 |
| WDR49 | 23 ( 6%) | 4 (2.5%) | 19 (9.1%) | 8.69e-03 | 3.69e-02 |
| SCN10A | 40 (11%) | 10 ( 6.2%) | 30 (14.4%) | 1.15e-02 | 4.37e-02 |
| ROBO1 | 29 ( 8%) | 5 ( 3.1%) | 24 (11.5%) | 2.92e-03 | 2.00e-02 |
| SLC12A7 | 21 ( 6%) | 8 (4.9%) | 13 (6.2%) | 6.56e-01 | 6.99e-01 |
| CDH18 | 21 ( 6%) | 2 (1.2%) | 19 (9.1%) | 1.01e-03 | 1.44e-02 |
| ROS1 | 25 ( 7%) | 5 (3.1%) | 20 (9.6%) | 1.27e-02 | 4.47e-02 |
| SGK223 | 22 ( 6%) | 2 (1.2%) | 20 (9.6%) | 5.64e-04 | 1.08e-02 |
| ADCY8 | 34 ( 9%) | 11 ( 6.8%) | 23 (11.1%) | 2.04e-01 | 2.68e-01 |
| TEX15 | 22 ( 6%) | 6 (3.7%) | 16 (7.7%) | 1.24e-01 | 1.85e-01 |
| BCL9L | 23 ( 6%) | 6 (3.7%) | 17 (8.2%) | 8.60e-02 | 1.37e-01 |
| AKAP6 | 27 ( 7%) | 9 (5.6%) | 18 (8.7%) | 3.16e-01 | 3.81e-01 |
| ITGB4 | 21 ( 6%) | 3 (1.9%) | 18 (8.7%) | 5.54e-03 | 2.83e-02 |
| SHANK1 | 19 ( 5%) | 3 (1.9%) | 16 (7.7%) | 1.56e-02 | 4.61e-02 |
| TIE1 | 22 ( 6%) | 5 (3.1%) | 17 (8.2%) | 4.65e-02 | 9.50e-02 |
| CHD5 | 26 ( 7%) | 5 ( 3.1%) | 21 (10.1%) | 1.25e-02 | 4.47e-02 |
| HLA-B | 19 ( 5%) | 2 (1.2%) | 17 (8.2%) | 3.24e-03 | 2.00e-02 |
| BAI1 | 22 ( 6%) | 4 (2.5%) | 18 (8.7%) | 1.40e-02 | 4.47e-02 |
| TRPC5 | 19 ( 5%) | 3 (1.9%) | 16 (7.7%) | 1.56e-02 | 4.61e-02 |
| ATP2B3 | 20 ( 5%) | 5 (3.1%) | 15 (7.2%) | 1.05e-01 | 1.62e-01 |
| KIAA2022 | 27 ( 7%) | 5 ( 3.1%) | 22 (10.6%) | 7.77e-03 | 3.49e-02 |
| MYH8 | 20 ( 5%) | 9 (5.6%) | 11 (5.3%) | 1.00e+00 | 1.00e+00 |
| CCDC178 | 19 ( 5%) | 2 (1.2%) | 17 (8.2%) | 3.24e-03 | 2.00e-02 |
| C20ORF26 | 19 ( 5%) | 3 (1.9%) | 16 (7.7%) | 1.56e-02 | 4.61e-02 |
| RASA1 | 22 ( 6%) | 5 (3.1%) | 17 (8.2%) | 4.65e-02 | 9.50e-02 |
| SOX7 | 21 ( 6%) | 7 (4.3%) | 14 (6.7%) | 3.71e-01 | 4.27e-01 |
| PNPLA7 | 19 ( 5%) | 5 (3.1%) | 14 (6.7%) | 1.55e-01 | 2.17e-01 |
| SEMA6D | 20 ( 5%) | 4 (2.5%) | 16 (7.7%) | 3.55e-02 | 7.74e-02 |
| POLE | 23 ( 6%) | 5 (3.1%) | 18 (8.7%) | 3.05e-02 | 7.31e-02 |
| ZNF521 | 31 ( 8%) | 11 (6.8%) | 20 (9.6%) | 3.52e-01 | 4.13e-01 |
| CILP2 | 20 ( 5%) | 4 (2.5%) | 16 (7.7%) | 3.55e-02 | 7.74e-02 |
| ZC3H4 | 21 ( 6%) | 5 (3.1%) | 16 (7.7%) | 7.03e-02 | 1.17e-01 |
| PLEKHA6 | 21 ( 6%) | 5 (3.1%) | 16 (7.7%) | 7.03e-02 | 1.17e-01 |
| PTPRU | 21 ( 6%) | 4 (2.5%) | 17 (8.2%) | 2.24e-02 | 5.76e-02 |
| COL5A2 | 20 ( 5%) | 3 (1.9%) | 17 (8.2%) | 9.33e-03 | 3.69e-02 |
| ARPP21 | 20 ( 5%) | 4 (2.5%) | 16 (7.7%) | 3.55e-02 | 7.74e-02 |
| EPHA6 | 28 ( 8%) | 8 (4.9%) | 20 (9.6%) | 1.13e-01 | 1.70e-01 |
| KLF3 | 19 ( 5%) | 4 (2.5%) | 15 (7.2%) | 5.56e-02 | 1.04e-01 |
| PCDHGA8 | 19 ( 5%) | 6 (3.7%) | 13 (6.2%) | 3.45e-01 | 4.07e-01 |
| HIVEP1 | 22 ( 6%) | 2 (1.2%) | 20 (9.6%) | 5.64e-04 | 1.08e-02 |
| AMPH | 20 ( 5%) | 7 (4.3%) | 13 (6.2%) | 4.92e-01 | 5.36e-01 |
| ATRX | 23 ( 6%) | 5 (3.1%) | 18 (8.7%) | 3.05e-02 | 7.31e-02 |
| LRFN5 | 19 ( 5%) | 6 (3.7%) | 13 (6.2%) | 3.45e-01 | 4.07e-01 |
| ARHGAP33 | 20 ( 5%) | 5 (3.1%) | 15 (7.2%) | 1.05e-01 | 1.62e-01 |
| WHSC1 | 19 ( 5%) | 2 (1.2%) | 17 (8.2%) | 3.24e-03 | 2.00e-02 |
| LRFN3 | 19 ( 5%) | 4 (2.5%) | 15 (7.2%) | 5.56e-02 | 1.04e-01 |
| CCNB3 | 21 ( 6%) | 3 (1.9%) | 18 (8.7%) | 5.54e-03 | 2.83e-02 |
| CNTN6 | 36 (10%) | 8 ( 4.9%) | 28 (13.5%) | 7.36e-03 | 3.48e-02 |
| CHRM2 | 20 ( 5%) | 8 (4.9%) | 12 (5.8%) | 8.19e-01 | 8.57e-01 |
| TRIM51 | 19 ( 5%) | 2 (1.2%) | 17 (8.2%) | 3.24e-03 | 2.00e-02 |
| ATP8B4 | 20 ( 5%) | 4 (2.5%) | 16 (7.7%) | 3.55e-02 | 7.74e-02 |
| SUPT6H | 21 ( 6%) | 4 (2.5%) | 17 (8.2%) | 2.24e-02 | 5.76e-02 |
| COL20A1 | 20 ( 5%) | 4 (2.5%) | 16 (7.7%) | 3.55e-02 | 7.74e-02 |
| IMPG2 | 22 ( 6%) | 4 (2.5%) | 18 (8.7%) | 1.40e-02 | 4.47e-02 |
| MDGA2 | 19 ( 5%) | 5 (3.1%) | 14 (6.7%) | 1.55e-01 | 2.17e-01 |
| AFF3 | 27 ( 7%) | 7 (4.3%) | 20 (9.6%) | 6.87e-02 | 1.17e-01 |
| MYO5A | 20 ( 5%) | 4 (2.5%) | 16 (7.7%) | 3.55e-02 | 7.74e-02 |
| MYH1 | 20 ( 5%) | 8 (4.9%) | 12 (5.8%) | 8.19e-01 | 8.57e-01 |
| CUL7 | 19 ( 5%) | 5 (3.1%) | 14 (6.7%) | 1.55e-01 | 2.17e-01 |
